# IL-17RA receptor signaling contributes to lung inflammation and parasite burden during *Toxocara canis* infection in mice

**DOI:** 10.3389/fimmu.2022.864632

**Published:** 2022-06-29

**Authors:** Thaís Leal-Silva, Camila de Almeida Lopes, Flaviane Vieira-Santos, Fabrício Marcus Silva Oliveira, Lucas Kraemer, Luiza de Lima Silva Padrão, Chiara Cássia Oliveira Amorim, Jorge Lucas Nascimento Souza, Remo Castro Russo, Ricardo Toshio Fujiwara, Luisa Mourão Dias Magalhães, Lilian Lacerda Bueno

**Affiliations:** ^1^ Laboratory of Immunology and Genomics of Parasites, Department of Parasitology, Institute of Biological Sciences, Federal University of Minas Gerais, Belo Horizonte, Brazil; ^2^ Faculdade de Medicina, Federal University of Minas Gerais, Belo Horizonte, Brazil; ^3^ Laboratory of Pulmonary Immunology and Mechanics, Department of Physiology and Biophysics, Institute of Biological Sciences, Federal University of Minas Gerais, Belo Horizonte, Brazil

**Keywords:** IL-17RA, toxocariasis, cytokines, helminth, inflammation, neutrophil

## Abstract

IL-17 is a cytokine produced by innate and acquired immunity cells that have an action against fungi and bacteria. However, its action in helminth infections is unclear, including in *Toxocara canis* infection. Toxocariasis is a neglected zoonosis representing a significant public health problem with an estimated seroprevalence of 19% worldwide. In the present study, we describe the immunopathological action of IL-17RA in acute *T. canis* infection. C57BL/6j (WT) and IL-17RA receptor knockout (IL-17RA-/-) mice were infected with 1000 *T. canis* eggs. Mice were evaluated 3 days post-infection for parasite load and white blood cell count. Lung tissue was harvested for histopathology and cytokine expression. In addition, we performed multiparametric flow cytometry in the BAL and peripheral blood, evaluating phenotypic and functional changes in myeloid and lymphoid populations. We showed that IL-17RA is essential to control larvae load in the lung; however, IL-17RA contributed to pulmonary inflammation, inducing inflammatory nodular aggregates formation and presented higher pulmonary IL-6 levels. The absence of IL-17RA was associated with a higher frequency of neutrophils as a source of IL-4 in BAL, while in the presence of IL-17RA, mice display a higher frequency of alveolar macrophages expressing the same cytokine. Taken together, this study indicates that neutrophils may be an important source of IL-4 in the lungs during *T. canis* infection. Furthermore, IL-17/IL-17RA axis is important to control parasite load, however, its presence triggers lung inflammation that can lead to tissue damage.

## Introduction

IL-17 is an inflammatory cytokine produced by several innate and adaptive immune cells such as Th17 lymphocytes, CD8 T cells, natural killer cells, γδ T cells, macrophages, neutrophils, eosinophils, and innate lymphoid cells (ILCs) (1–3). IL-17 family cytokines mediate effects at the molecular level by binding to their receptors, known as IL-17R family receptors (IL-17RA - IL-17RE), which have unique structural characteristics and trigger signaling events (4). IL-17A and IL-17F are homonymous cytokines of the Th17 lineage, which can form IL-17A–IL-17F heterodimers and are the best characterized cytokines of the IL-17 lineage. The cytokines IL-17A, IL-17F and IL-17A–IL-17F signal through the same receptor subunits, IL-17RA and IL-17RC, which together form a heteromeric complex (4, 5). The inflammatory capacity of IL-17 is related to the recruitment of immune cells such as neutrophils and monocytes and synergistic action with other cytokines such as TNF, IL-1β, IFN-γ, GM-CSF, IL-22 (5).

The IL-17 presents an important response against extracellular bacteria and fungi (5). However, the role of IL-17 in helminth infections is still controversial and appears to be pathogen-specific. Studies in animal models infected with *Schistosoma* spp. indicated that IL-17 contributed to the pathogenesis of liver fibrosis and increased granulomatous inflammation in the lung and liver (6–8). In *Ascaris suum* infection, the systemic polarized Th2/Th17 immune response appears to be crucial to control larval migration after multiple exposures to *Ascaris* (9). Using a model of lung infection by *Nippostrongylus brasiliensis*, it was reported that IL-17 and neutrophilic inflammation limited parasite survival but caused increased lung injury (10). Furthermore, it was demonstrated that IL-4R signaling controlled IL-17 elevations, increased IL-10 and stimulated the development of M2 cells, contributing to the resolution of tissue damage, showing that the Th2 response can contribute to the acute healing of wounds during helminth infection (11). Another study reported that IL-17A is an important regulator of type 2 pulmonary immunity in *N. brasiliensis* infection. IL-17A supports the development of type 2 response through IFN-γ suppression, however, in the later phase, IL-17A limited excessive type 2 responses, proposing a feedback mechanism (12). In *Toxocara canis* infection, studies have shown that IL-17 is increased in serum and lung tissue, but its role during infection has not yet been elucidated (13, 14).

Toxocariasis is a neglected zoonosis whose principal etiological agent is *T. canis*. It is estimated that the worldwide seroprevalence of toxocariasis is around 19%, with the highest rates being associated with countries with higher temperature and humidity, lower-income levels, and lower human development index (11, 15). Humans become infected by accidentally ingesting eggs containing the infecting larvae, hatching the intestine, penetrating the intestinal mucosa, and migrating to various organs, including the lungs, where they trigger an inflammatory response with eosinophilia and increased production of cytokines and specific antibodies (16–18).

In recent years, the number of studies on the immune response triggered by *T. canis* has increased (9, 10, 13, 14), however, the immunological mechanisms involved in protection and injury during infection are still poorly understood. In this context, the present study aimed to describe the immunopathological role of IL-17RA in acute *T. canis* infection. Our results revealed that IL-17/IL-17RA axis is important for controlling the pulmonary larval load, and this fact may be related to the increase in IL-6 and the frequency of IL-4 producing neutrophils. However, its presence increases pulmonary inflammation and can trigger lung tissue damage.

## Methods

### Parasites

Adult *T. canis* worms were collected from feces of naturally infected puppies treated with anthelmintics (Drontal Puppy, São Paulo, Brazil) at a dosage of 1mL/kg. Puppies were kept at the Zoonoses Control Center in Belo Horizonte, Minas Gerais, Brazil. Adult worms were kept in water and taken to the Laboratory of Immunology and Genomics of Parasites at the Federal University of Minas Gerais to be processed. Eggs were isolated from the uterus of adult female worms by mechanical maceration, purified by filtration on 100 µm nylon filters, placed in culture flasks with 50 mL of 0.2 M sulfuric acid, and kept in a BOD incubator at 26°C. After 6 weeks of cultivation, at the peak of larval infectivity, fully embryonated eggs were used for experimental infections (13).

### Mice

Female C57BL/6j or IL-17RA^-/-^ mice at approximately 8 weeks of age were used for this study. Mice genetically deficient for the IL-17RA receptor (IL-17RA^-/-^) were acquired at the Special Mouse Breeding Center of the Faculdade de Medicina de Ribeirão Preto (USP), and wild-type mice (WT) C57BL/6j were obtained from the animal facility of the Federal University of Minas Gerais. During the experimental period, mice were fed with filtered water and commercial chow (Nuvilab Cr-1, Nuvital Nutrients, Brazil) ad libitum. Mice were maintained at the Animal Facility of the Department of Parasitology of the Federal University of Minas Gerais under controlled temperature conditions (24 ± 1°C) and lighting (12-hour light-dark cycle).

### Experimental design and *T. canis* infection

**Figure 1:**
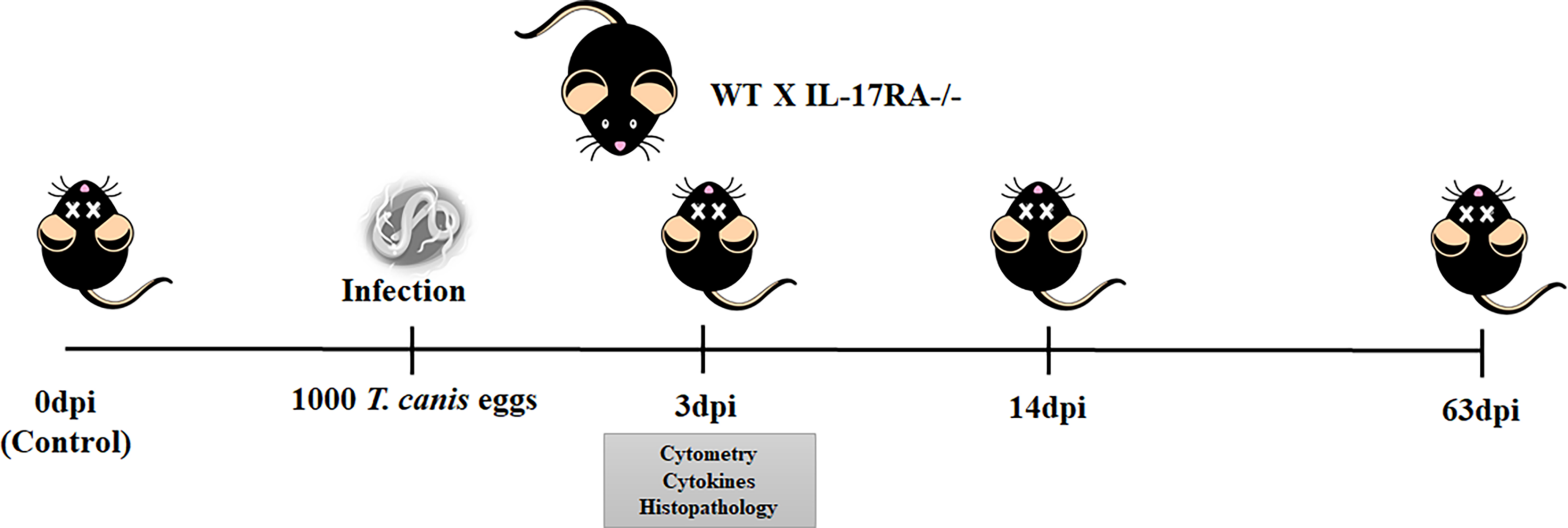
Experimental design of *T. canis* infection in mice. WT and IL-17RA-/- mice were infected with 1000 eggs of *T. canis* and euthanized with 0dpi, 3dpi, 14dpi, and 63dpi. With 3dpi, cytometry, cytokine, and histopathology analysis were performed.

WT and IL-17RA^-/-^ mice were randomized and sacrificed on day 0 (control group), 3, 14, and, 63 days post-infection (dpi), as shown in **Figure 1**. Fifty mice of each strain were used for performing all methodologies, distributed as follows: 6 mice for each dpi to assess parasite burden and leukocyte profile (0dpi, 3dpi, 14dpi, and 63dpi), 6 mice for flow cytometry (0dpi and 3dpi), and 7 mice for histopathology and cytokine evaluation (0dpi and 3dpi). All mice were sacrificed with a lethal injection of xylazine/ketamine (8.5 mg/kg and 130 mg/kg).

For *T. canis* infection, prior to inoculation, embryonated eggs were incubated with 5% (v/v) sodium hypochlorite solution in an incubator (37°C and 5% CO_2_) for 1 hour and 40 minutes to break up the outer layer of eggs and, therefore, to facilitate the hatching of larvae *in vivo*. After incubation, eggs were resuspended and washed with PBS 3 times. Mice from infected groups were inoculated by oral gavage with 0.2 ml of the solution containing 1000 embryonated eggs.

### Parasitological analysis

Parasite burdens of infected mice were assessed by counting the total number of larvae recovered in the liver, lung, and brain with 3dpi, 14dpi, and 63dpi. Each tissue was collected, punctured with surgical scissors, and placed in a modified Baermann apparatus for 4 h in PBS at 37°C and 5% CO_2_. The larvae recovered in the pellet of the apparatus were fixed in 4% formalin and quantified by light microscopy (14).

### Leukocyte analysis

500μL of blood was collected by cardiac puncture and transferred to tubes containing the anticoagulant EDTA (Vacuplast, Brazil). Global leukocyte counts were performed using a Bio-2900 Vet automated hematology counter. Blood smears were stained with Panotic (Laborclin, Brazil) for differential leukocyte counts, and 100 leukocytes were differentiated under a light microscope.

### Protein and hemoglobin in bronchoalveolar lavage

Mice were anesthetized, and a 1.7 mm catheter was inserted into the trachea. One milliliter of PBS was washed twice through the catheter to collect the bronchoalveolar lavage fluid. The lavage fluid was centrifuged at 3000 g for 10min, and the supernatants were used for hemoglobin and total protein quantification.

Hemoglobin was quantified to assess alveolar hemorrhage present in the BAL using the Hemoglobin K023-1 kit (Bioclin Quibasa, Brazil). Concentration was determined spectrophotometrically by measuring absorbance at 540 nm. Hemoglobin (Hb) concentration was expressed in g/dL of Hb per ml of BAL. Quantitation of total protein was determined by the BCA Protein Assay kit (Thermo Scientific, USA). Results were expressed as μg of total protein per ml of BAL.

### Cytokine profiles

Pulmonary cytokines were evaluated at 3dpi in infected and uninfected mice. For that, the right lung of each animal was removed and homogenized (TissueLyser LT- Qiagen, German) in extraction solution (0.4 M NaCl, 0.05% Tween 20, 0.5% BSA, 0.1 mM phenylmethylsulfonyl fluoride, 0.1 mM benzethonium chloride, 10 mM EDTA and 20 IU aprotinin A) at a rate of 1 mL per 100 mg of lung tissue. The homogenates were centrifuged at 800 × g for 10 min at 4°C, and the supernatant was collected and stored at -80°C for cytokine quantification. Levels of TNF-α, IL-1β, IL-6, IL-12/IL-23p40, IL-17A, IL-4, IL-5, IL-33, IL-13, IL-10, and TGF-β were tested by an ELISA sandwich kit (R&D Systems, USA) according to the manufacturer’s instructions. The absorbance of the samples was determined in a Versa Max ELISA microplate reader (Molecular Devices, USA) at a wavelength of 492 nm.

### Histopathological analysis

The left lobe of the lungs was removed from mice infected with 3dpi and from uninfected 0dpi. The organs were fixed in a 4% formalin solution, gradually dehydrated in ethanol, cleared in xylene, and embedded in paraffin blocks that were cut 4–5 micrometers thick and fixed on a microscope slide. Lung tissue slides were stained with hematoxylin and eosin. All histopathological analyses were performed blindly.

For the airway inflammation score, 10 random images per animal were captured at 20X magnification as described in **Supplemental Table 1** and analyzed for perivascular inflammation, peribronchial inflammation, parenchymal damage, hemorrhage, and nodular aggregates formation.

### Phenotypic analysis by flow cytometry

Multiparametric flow cytometric analyses were performed in BAL and peripheral blood of WT and IL-17RA^-/-^ mice uninfected (0dpi) and infected with 3dpi to characterize the cell population and observe the cytokines secreted by each cell type.

In the BAL, all the following Bioscience® markers were used: CD4 (Fitc, clone H129.19, 1:100), IL-4 (PE, clone 11B11, 1:50), Siglec F (PeTxRed, clone E50-2440, 1:400), CD45 (PeCy7, clone 30-F11, 1:800), CD11c (APC, clone N418, 1:800), IL-17 (APCCy7, clone TC11-18H10, 1:50), I-A/I-E (MHCII) (BV480, clone M5/114.15.2, 1:50) IL-10 (BV421, clone JES5-16E3, 1:50), Ly6G (BV570, clone 1A8, 1:200).

In peripheral blood, two panels were used, in the first, the following Bioscience® markers were used: CD69 (Fitc, clone H1.2F3, 1:500) IL-4 (PE, clone 11B11, 1:50), Siglec F (PeTxRed, clone E50-2440, 1:400) CD4 (PeCy5, clone RM4-5, 1:800), CD27 (PeCy7, clone LG3A10, 1:200), CD8 (APC, clone 53-6.7, 1:800), IL-17 (APCCy7, clone TC11-18H10, 1:50), Viability (Fixable viability stain 700, 1:1000), I-A/I-E (MHCII) (BV480, clone M5/114.15.2, 1:50), TNF-α (BV421, clone MP6- XT22, 1:200), Ly6G (BV570, clone 1A8, 1:200).

In the second panel for monocyte identification, the following Bioscience^®^ markers were used: I-A/I-E (MHCII) (Fitc, clone AF6-120.1, 1:50), Ly6C (PE, clone AL-21, 1:800), CD11b (PeCy5, clone M1/70, 1:200), TNF-α (BV421, clone MP6-XT22, 1:200), CX3CR1 (BV605, clone SA011F11, 1:200), Viability (Fixable viability stain 700, 1:1000).

BAL and peripheral blood cells were incubated with brefeldin (BD Biosciences^®^) at a concentration of 1μg/ml at 5% CO_2_ for 4 hours at 37°C. After brefeldin incubation, cells were incubated with ACK lysis buffer for 10 minutes (1x for BAL and 3x for peripheral blood). After each lysis cycle, cells were washed with PBS and centrifuged at 300 G, 8 minutes, 4°C. Once the samples were free of erythrocytes, cells were counted in Neubauer chamber using Trypan blue and incubated with the viability dye (BD Bioscience®, Fixable viability stain 700, 1:1000) for 10 min at 4°C. After incubation, cells were washed by centrifugation at 300 G, 8 minutes, 4°C PBS. After Live/Dead staining, cells were incubated with a 20μl mix of surface antibodies containing 10% normal mice serum for 15 minutes at 4°C and washed with PBS by centrifugation at 300 G, 8 minutes, 4°C. Samples were then fixed with 2% PFA for 20min at room temperature. After fixation, cells were washed twice with PBS and permeabilized by incubation for 15min with a 0.5% saponin solution and proceeded to intracellular staining. 40μl of intracellular antibody mix were incubated for 30 min at room temperature. After intracellular staining, cells were washed twice with 0.5% saponin solution and resuspended in PBS. Samples were acquired in LSR Fortessa (BD Biosciences, USA) and analyzed with FlowJo software (Tree Star, Ashland, OR). Data analysis was followed by dimensionality reduction and visualization by t-Distributed Stochastic Neighbor Embedding (tSNE) using Cytofkit (23) and rPhenograph to group cells into clusters according to their similarity of activation/expression molecules and cytokines and rPhenograph to group cells into clusters according to their similarity of activation/expression molecules and cytokines. Each experimental group in tSNE analysis represent 6 mice with input of the same number of cells. The gating strategy is illustrated in **Supplementary Figure 1** and the summary of all markers used in flow cytometry are in the **Supplemental Table 2**.

### Statistical analysis

Statistical analysis was performed using Prism 8.0 software (GraphPad Inc, USA). Initially, the Grubbs test was used to detect possible outlier values. Followed by the Shapiro-Wilk test was performed to assess the normality of the variables. The comparison between two groups was performed using the Student’s T test or the Mann-Whitney test according to the normality test. The evaluation between three or more groups was performed using the Analysis of Variance (ANOVA) or Kruskal-Wallis test, followed by Tukey’s or Dunn’s post-test, respectively, according to the data distribution. In all analyses, the results with a value of p<0.05 were considered statistically significant.

## Results

### The absence of the IL-17RA receptor increases the pulmonary parasite load and induces leukocytosis

**Figure 2:**
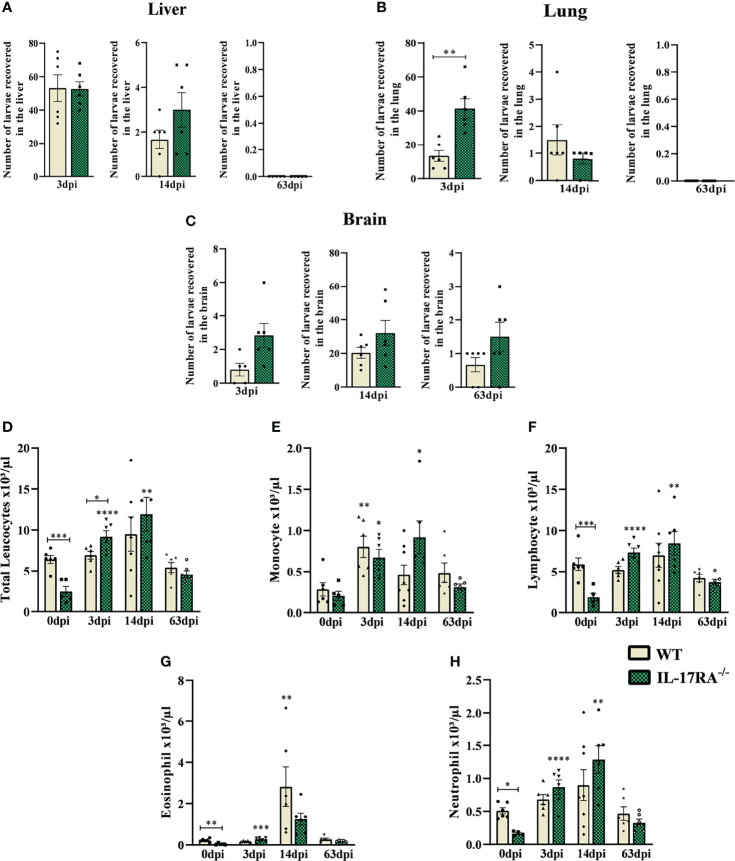
Parasite load and blood leukocyte count from WT and IL-17RA-/- mice during *T. canis* infection. Parasite burden was assessed by counting the total number of larvae in the liver, lung, and brain after 3, 14, and 63 dpi. Blood was collected by cardiac puncture for leukocyte count. **(A)** The number of larvae recovered from the liver in 3 days post-infection (dpi), 14dpi and 63dpi; **(B)** Number of larvae recovered from lung in 3dpi,14dpi and 63dpi; **(C)** Number of larvae recovered from the brain in 3dpi,14dpi and 63dpi; **(D)** Total leukocytes count; **(E)** Monocytes count; **(F)** Lymphocytes count; **(G)** Eosinophils count; **(H)** Neutrophils count. Statistical analyses were performed between each strain with its uninfected group (0dpi), represented by the asterisk without the bar, and between the two strains at the same time of infection, represented by the asterisk with the bar. Non-significant differences were not reported in the bar. Results represent the mean ± Standard error of the mean (SEM), Cream bars represent WT mice and green bars represent IL17RA-/- mice, *p<0.05, **p<0.01, ***p<0.001, ****p<0.0001. According to the normality test, Student’s t test was used in graphs A for 3dpi and 14dpi, B for 3dpi and C for 3dpi and 14dpi in the comparison between WT and IL-17RA-/- on each day of infection (dpi). The Mann-Whitney test was used in graphs A for 63dpi, B for 14dpi and 63dpi and C for 63dpi in comparing WT and IL-17RA-/- on each day of infection (dpi). One-way ANOVA test followed by Tukey’s test was used on D, E, F, G, H graphs. N = 6 mice per experimental group.

To assess the response of IL-17RA in *T. canis* infection, the larvae recovery in the liver, lungs, and brain tissues was performed (**Figures 2A–C**). A significant increase in larvae in lung tissue after 3dpi was observed in IL-17RA^-/-^ mice compared to WT mice.

To assess the systemic effects of *T. canis* infection, a differential blood cell count was performed in the acute and chronic phases of the infection (**Figures 2D–H**). The results showed that IL-17RA^-/-^ mice naturally have fewer total leukocytes than WT (p = 0.0008), with lower numbers of eosinophils (p = 0.0025), neutrophils (p = 0.0443), and lymphocytes (p = 0.0005). However, there was an increase in leukocytes in IL-17RA^-/-^ mice in the acute phase of infection (3dpi) (p = 0.0486), while leukocyte counts remained stable in WT mice (**Figure 2D**). At 3dpi, an increase in monocytes was observed in both strains, and this increase remained up to 14dpi in IL-17RA^-/-^ mice compared to their control (**Figure 2E**). Also, in IL-17RA^-/-^ mice, an increase in lymphocytes and neutrophils was observed at 3dpi and 14dpi (**Figures 2F, H**) compared to the uninfected. In WT mice, we observed the eosinophilia peak at 14dpi when compared to its control (**Figure 2G**).

### *T. canis* infection induces increased TNF-**α** expression by non-classical monocytes regardless of the presence of IL-17RA receptor

**Figure 3:**
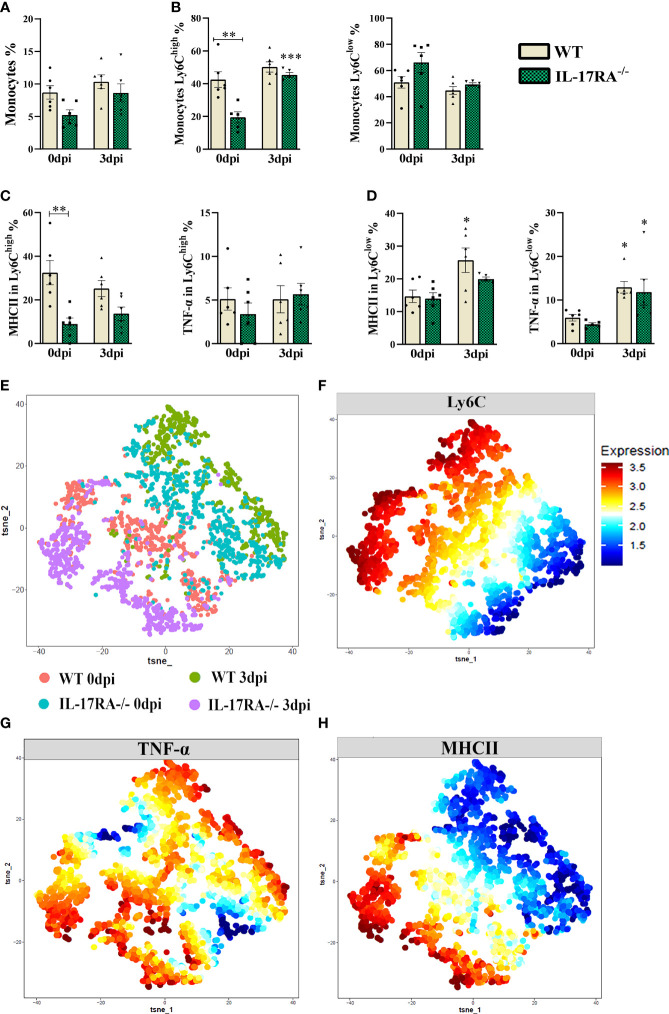
TNF-α and MHCII expression in Ly6C^low^ and Ly6C^high^ monocytes populations from WT and IL-17RA-/- mice during *T. canis* infection. (A) Bar show frequency of total monocytes; (B) Bar show frequency of classic monocytes Ly6C^high^ (left) and non-classical monocytes Ly6C^low^ (right); (C) Bar show frequency of MHC in classic monocytes Ly6C^high^ (left) and TNF-α classic monocytes Ly6C^high^ (right); (D) Bar show frequency of MHC in classic monocytes Ly6C^low^ (left) and TNF-α classic monocytes Ly6C^low^ (right); (E) Unsupervised high dimensional analysis of flow cytometry data (tSNE), gated on LiveCD11b+CX3CR1+ monocyte from combined data of blood obtained from WT 0dpi (red), WT 3dpi (green), IL17RA-/- 0dpi (blue) and IL17RA-/- 3dpi (purple); (F) Expression levels of Ly6C markers; (G) Expression map TNF-α; (H) Expression map MHCII. Statistical analyses were performed between each strain with its uninfected group (0dpi), represented by the asterisk without the bar, and between the two strains at the same time of infection, represented by the asterisk with the bar. Non-significant differences were not reported in the bar. Data represented as mean ± SEM, *p < 0.05, **p < 0.01, ***p < 0.001. For data that passed the normality test [A, B (left), C (right) and D (left)] One-way ANOVA test followed by the Tukey test was used, while non-parametric data B (right), C (left) and D (right) were used Kruskal-Wallis test followed by the Dunn test. N = 6 animals per group with two independent experiments.

Monocytes from peripheral blood were gated as LiveCD11b+CX3CR1+ and subdivided as classical (Ly6C^high^) and non-classical (Ly6C^low^) by multiparametric flow cytometry (**Supplementary Figures 1** and **2**). No difference was observed in the total monocyte population after infection in WT or IL-17RA^-/-^ mice; however, when we analyzed the percentage of subpopulation of classical monocytes (Ly6C^high^), we observed that uninfected WT 0dpi mice showed a higher percentage of Ly6C^high^ monocytes (p = 0.0013) and MHCII in Ly6C^high^ (p = 0.0065) compared to IL-17RA^-/-^ (**Figures 3A–E**).

The results showed that infected WT mice increased the percentage of MHCII in non-classical monocytes compared to their uninfected group (WT 0dpi) and that infection by *T. canis*, regardless of the presence of IL-17RA^-/-^, induced an increase in TNF-α expression in non-classical monocytes when compared to their respective uninfected groups.

The mean fluorescence intensity (MFI) shown by tSNE (**Figures 3E–H**), indicated that there is greater expression of MHCII in the WT 0dpi and IL-17RA^-/-^ 3dpi groups, whereas the expression of TNF-α indicated to be greater in the WT 0dpi groups and in the infected groups.

### The IL-17RA deficiency increases the frequency of eosinophils in peripheral blood during *T. canis* infection

**Figure 4:**
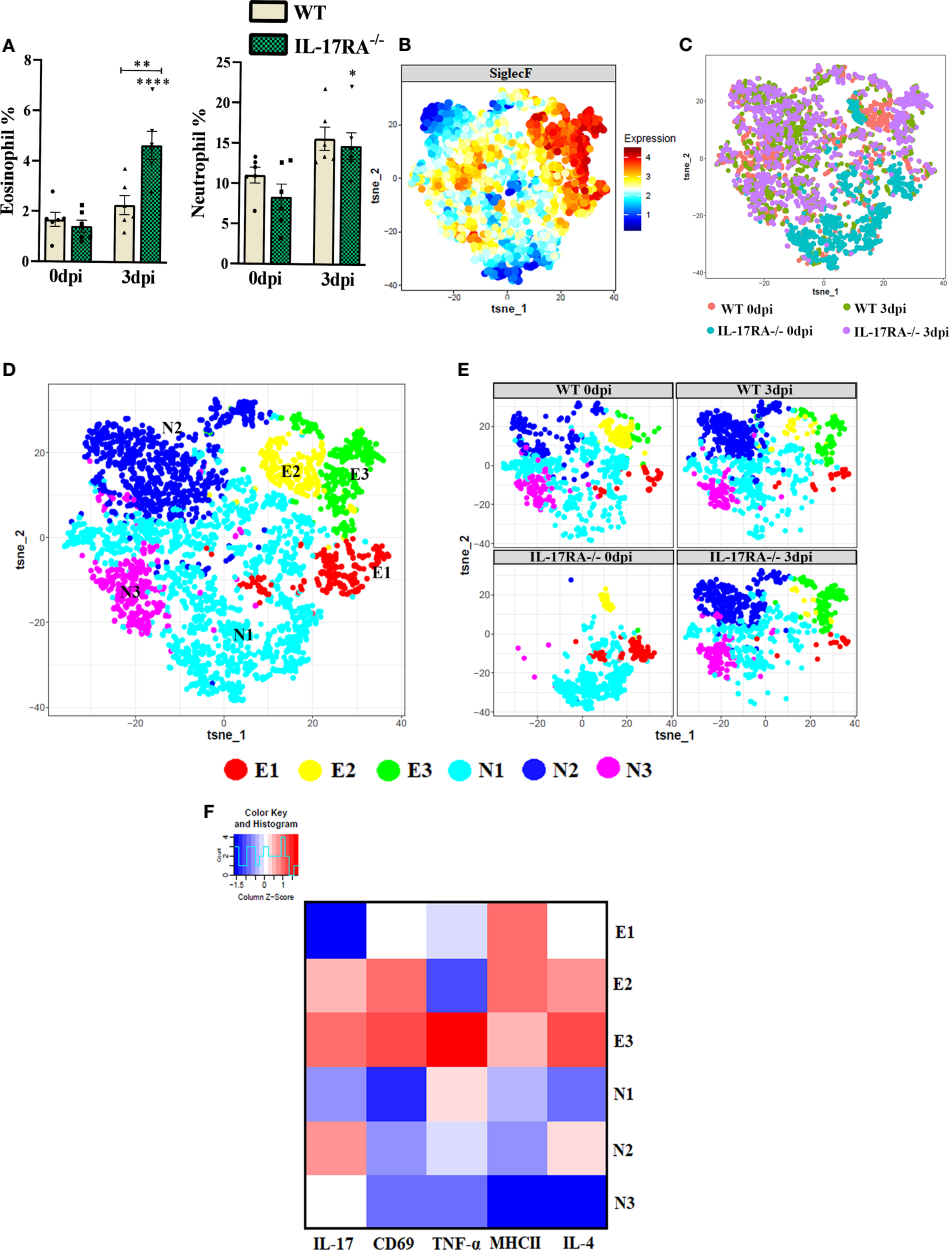
Analysis of neutrophil and eosinophil clusters of peripheral blood by cytometry in WT and IL-17RA-/- mice during *T. canis* infection. (A) Bar show frequency of total eosinophils (left) and neutrophils (right); (B) Expression levels of SiglecF; (C) Unsupervised high dimensional analysis of flow cytometry data (tSNE), gated on SiglecF+ eosinophils and SiglecF- and Ly6G+ neutrophils from combined data of blood obtained from WT 0dpi (red), WT 3dpi (green), IL17RA-/- 0dpi (blue) and IL17RA-/- 3dpi (purple); (D) tSNE of “C” representing cluster analysis by rPhenograph, eosinophils “E” and neutrophils “N”; (E) tSNE of “C” depicting neutrophil and eosinophil clusters per group; (F) Heat Map with cluster marker expression. Statistical analyses were performed between each strain with its uninfected group (0dpi), represented by the asterisk without the bar, and between the two strains at the same time of infection, represented by the asterisk with the bar. Non-significant differences were not reported in the bar. Data represented as mean ± SEM, *p < 0.05, **p < 0.01, ***p < 0.001. According to the normality test, One-way ANOVA test followed by the Tukey test was used. N = 6 animals per group with two independent experiments.

Peripheral blood cytometry was performed to evaluate eosinophils (LiveSiglecF+) and neutrophils (LiveSiglecF-Ly6G+) (**Supplementary Figures 1** and **3**). Infection with *T. canis* led to increased frequency of both eosinophils and neutrophils in IL-17RA^-/-^ mice compared to IL-17RA^-/-^ 0dpi (**Figure 4A**). When comparing the two strains, a higher frequency of eosinophils was demonstrated in the IL-17RA^-/-^ 3dpi groups compared to the WT 3dpi (p = 0.0016). After separating eosinophils and neutrophils and identifying their presence in all groups (**Figures 4B, C**), in the dimensional reduction analysis, 3 eosinophils (E1-E3) and 3 neutrophil clusters (N1-N3) were clustered (**Figures 4D, E**). When observing the eosinophils, we observed that the E1 that had the highest expression of MHCII was present in all groups, the E2 present mainly in the WT 0dpi group had the highest expression of CD69, MHCII and IL-4 and the E3 present mainly in the infected groups had the highest expression of IL-17, IL-4 TNF-α and CD69 (**Figure 4F**).

When we analyzed the neutrophil clusters, we observed that the N1 present in all groups had a higher expression of TNF-α, the N2 present mainly in the infected groups showed the highest expression of IL-17 and IL-4, while the N3 present in all groups except for WT 0dpi, showed low expression of all markers used. Thus, we can infer that *T. canis* infection can induce a higher frequency of eosinophils expressing IL-17, TNF-α, IL-4 and CD69 and neutrophils expressing IL-17 and IL-4.

It is important to mention that the clusters reflect only the patter of expression of the above stated markers and do not reflect different development stages of eosinophils or neutrophils.

### In the acute phase of *T. canis* infection, no difference in the CD4 and CD8 T cell population was observed

**Figure 5:**
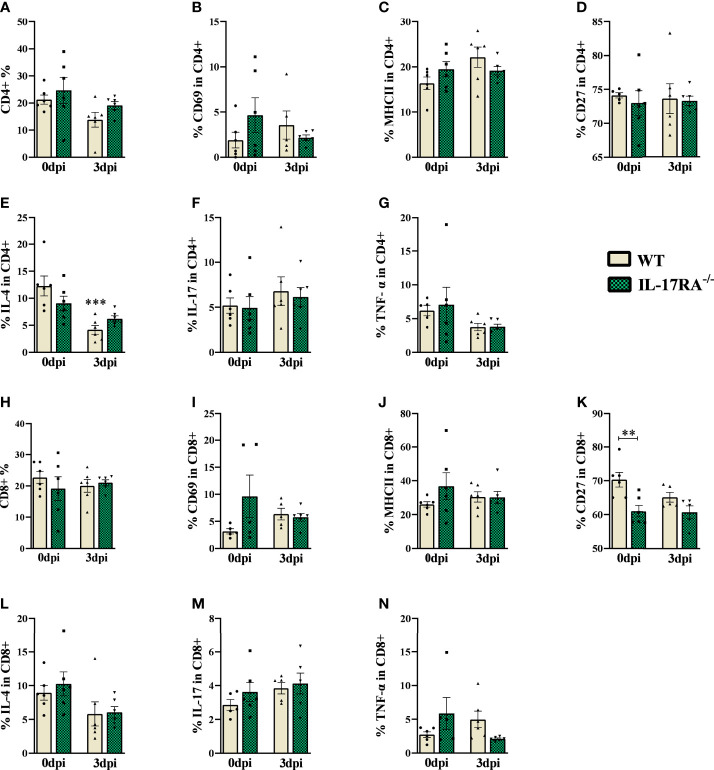
Flow cytometry analysis of TCD4 and TCD8 lymphocytes of peripheral blood in WT and IL-17RA-/- mice during *T. canis* infection. **(A)** Bar show frequency of total CD4 T lymphocytes; **(B)** Bar show frequency of CD69 in CD4 T lymphocytes; **(C)** Bar show frequency of MHCII in CD4 T lymphocytes; **(D)** Bar show frequency of CD27 in CD4 T lymphocytes; **(E)** Bar show frequency of IL-4 in CD4 T lymphocytes; **(F)** Bar show frequency of IL-17 in CD4 T lymphocytes; **(G)** Bar show frequency of TNF-α in CD4 T lymphocytes; **(H)** Bar show frequency of total CD8 T lymphocytes; **(I)** Bar show frequency of CD69 in CD8 T lymphocytes; **(J)** Bar show frequency of MHCII in CD8 T lymphocytes; **(K)** Bar show frequency of CD27 in CD8 T lymphocytes; **(L)** Bar show frequency of IL-4 in CD8 T lymphocytes; **(M)** Bar show frequency of IL-17 in CD8 T lymphocytes; **(N)** Bar show frequency of TNF-α in CD8 T lymphocytes. Statistical analyses were performed between each strain with its uninfected group (0dpi), represented by the asterisk without the bar, and between the two strains at the same time of infection, represented by the asterisk with the bar. Non-significant differences were not reported in the bar. Data represented as mean ± SEM, **p < 0.01, ***p < 0.001. According to the normality test, One-way ANOVA test followed by the Tukey test was used. N = 6 animals per group with two independent experiments.

To differentiate between CD4+ T lymphocyte and CD8+ T lymphocyte populations, we used the CD4 and CD8 markers (**Figure 5** and **Supplementary Figure 1**). When analyzing the frequencies of these populations, we did not observe statistical differences between the experimental groups (**Figures 5A–N**).

Regarding the markers and cytokines used for CD4 and CD8 lymphocytes, we observed that infected WT mice reduced the frequency of IL-4 compared to their control, while the IL-17RA^-/-^ 0dpi mice naturally showed a reduction in the frequency of CD27 compared to WT 0dpi. We did not observe significant differences between the infected groups.

### The IL-17RA receptor deficiency leads to increased IL-6 levels in the lungs after *T. canis* infection

**Figure 6:**
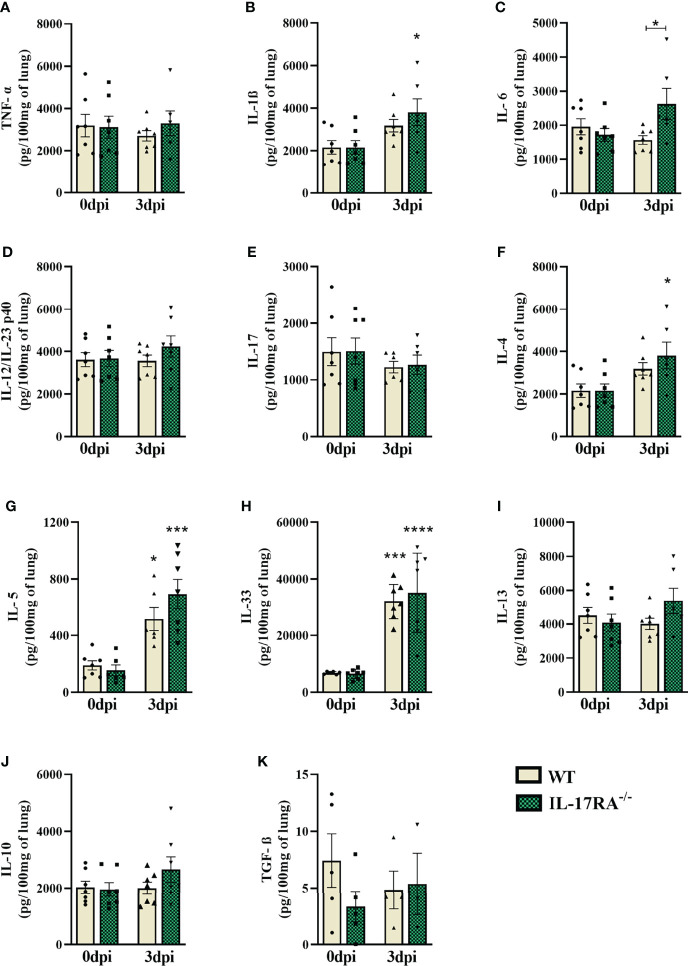
Cytokine profile in the lung parenchyma of WT and IL-17RA-/- mice during *T. canis* infection. The cytokines present in the lung parenchyma were measured by ELISA. **(A)** TNF-α; **(B)** IL-1β; **(C)** IL-6; **(D)** IL-12/IL-23p40; **(E)** IL-17; **(F)** IL-4; **(G)** IL-5; **(H)** IL-33; **(I)** IL-13; **(J)** IL-10; **(K)** TGF-β. Statistical analyses were performed between each strain with its uninfected group (0dpi), represented by the asterisk without the bar, and between the two strains at the same time of infection, represented by the asterisk with the bar. Non-significant differences were not reported in the bar. Results represent the mean ± SEM, Cream bars represent WT mice and green bars represent IL17RA-/- mice, *p < 0.05, ***p < 0.001, ****p < 0.0001. According to the normality test, One-way ANOVA test followed by Tukey test was used. N = 7 mice per experimental group.

For immunological analysis of the lung parenchyma in the acute phase of infection, cytokines from Th1, Th2, Th17, and Treg responses were measured by ELISA (**Figures 6A–K**). Infected WT mice showed an increase in Th2 cytokines (IL-5 and IL-33) compared to uninfected controls (0dpi). On the other hand, IL-17RA^-/-^ mice showed an increase in IL-1ß, IL-4, IL-5, and IL-33. When comparing the two strains, it was observed that IL-17RA^-/-^ mice increased the concentration of IL-6 after infection (p = 0.0481). Thus, these results demonstrate that in *T. canis* infection, the absence of the IL-17RA receptor favors an increase in IL-1ß, IL-4, and IL-6 in the lung parenchyma.

### IL-17RA receptor deficiency increases the frequency of neutrophils expressing IL-4 in bronchoalveolar lavage during *T. canis* infection

**Figure 7:**
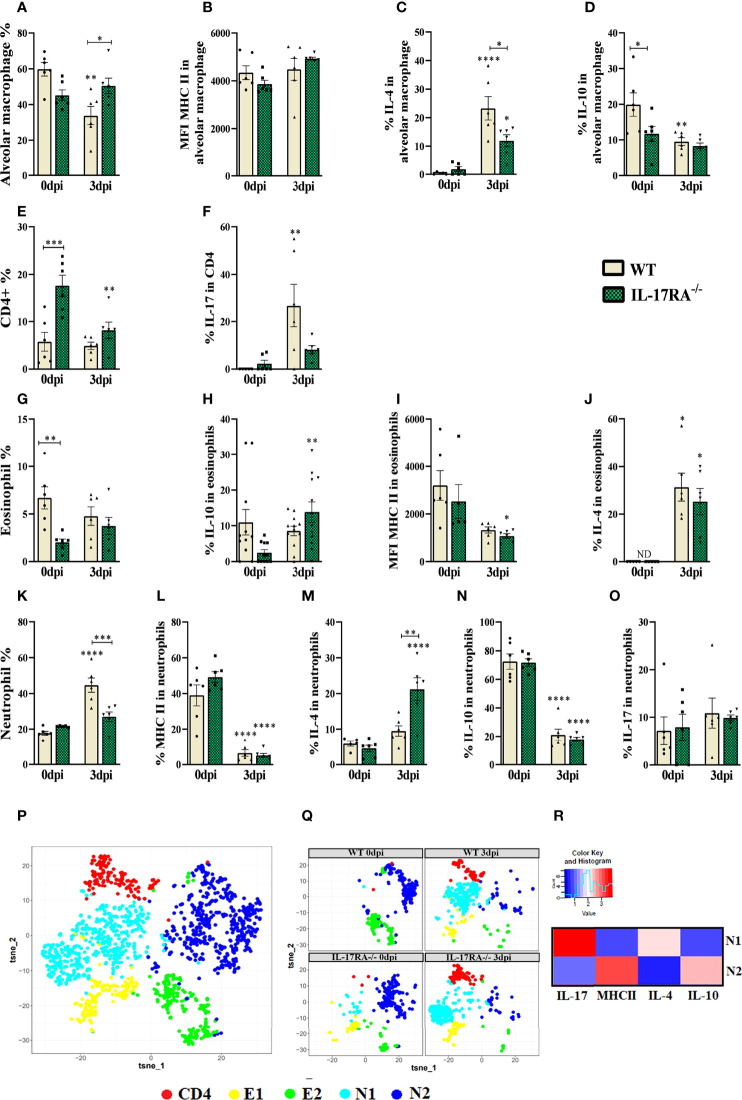
Flow Cytometry of bronchoalveolar lavage (BAL) analysis of leukocyte populations, alveolar macrophages, eosinophils, CD4+ and neutrophils in WT and IL-17RA-/- mice during *T. canis* infection. **(A)** The bar shows the frequency of alveolar macrophage; **(B)** The bar shows the mean intensity of fluorescence (MFI) of MHCII in alveolar macrophage; **(C)** The bar shows the frequency of IL-4 in alveolar macrophage; **(D)** The bar shows the frequency of IL-10 in alveolar macrophage; **(E)** The bar shows the frequency of CD4 T lymphocytes; **(F)** The bar shows the frequency of IL-17 in CD4 T lymphocytes; **(G)** The bar shows the frequency of eosinophils; **(H)** The bar shows the frequency of IL-10 in eosinophils; **(I)** The bar shows the MFI of MHCII in eosinophils; **(J)** The bar shows the frequency of IL-4 in eosinophils. ND= not-detected; **(K)** The bar shows the frequency of neutrophils; **(L)** The bar shows the frequency of MHCII in neutrophils; **(M)** The bar shows the frequency of IL-4 in neutrophils; **(N)** The bar shows the frequency of IL-10 in neutrophils; **(O)** The bar shows the frequency of IL-17 in neutrophils; **(P)** tSNE representing cluster analysis by rPhenograph, eosinophils “E”, neutrophils “N” and CD4 T lymphocytes “CD4”; **(Q)** tSNE of “P” depicting eosinophils, neutrophils and CD4 T lymphocytes clusters per group; **(R)** Heat Map with neutrophils cluster marker expression. Statistical analyses were performed between each strain with its uninfected group (0dpi), represented by the asterisk without the bar, and between the two strains at the same time of infection, represented by the asterisk with the bar. Non-significant differences were not reported in the bar. Data represented as mean ± SEM, * p < 0.05, ** p < 0.01, *** p < 0.001, **** p < 0.0001. For data that passed the normality test **(A–E, G, K–N)** One-way ANOVA test followed by the Tukey test was used, while non-parametric data **(F, H, I, J, O)** were used Kruskal-Wallis test followed by the Dunn test. N = 6 animals per group with two independent experiments.

BAL cytometry was performed to evaluate alveolar macrophages (LiveCD45+SiglecF+CD11c+), eosinophils (LiveCD45+CD11c-SiglecF+), neutrophils (LiveCD45+CD11c-SiglecF-Ly6G+) and CD4+ T lymphocytes (LiveCD45+low granularityCD4+) (**Figure 7**). When analyzing the frequencies of cells in uninfected mice, we observed a reduction in eosinophils (p = 0.0074) and an increase in CD4+ T lymphocytes (p = 0.0006) in IL-17RA^-/-^ mice compared to WT mice. In infected groups, we observed increased alveolar macrophages (p = 0.0460) and decreased neutrophils (p = 0.0005) in IL-17RA^-/-^ mice compared to WT mice.

When we analyzed the expression of cytokines in alveolar macrophages, we observed that naturally IL-17RA^-/-^ mice have a reduction in IL-10 compared to WT (p = 0.0470), and that during *T. canis* infection there is a reduction in IL-10 expression in these animals. Our results also showed that during *T. canis* infection there is an increase in IL-4 expression in these macrophages, being lower in IL-17RA^-/-^ 3 dpi mice compared to WT 3dpi (p = 0.0156) (**Figures 7A–D**).

Dimensional reduction analysis was able to identify 1 CD4+ T lymphocyte cluster, 2 eosinophil clusters (E1 and E2) and 2 neutrophil clusters (N1 and N2) (**Figures 7P, Q**). When analyzing the frequency of CD4+ cells, it was observed that IL-17RA^-/-^ 0dpi mice showed an increase in the frequency of CD4 T lymphocytes compared to WT 0dpi and that after infection there was a reduction in the frequency of these cells in the BAL in IL-17RA^-/-^ mice. It was also possible to observe an increase in the expression of IL-17 in these cells in WT 3dpi mice compared to their respective control group (**Figures 7E, F**).

Regarding eosinophils, we observed an increase in IL-10 in WT 3dpi and IL-4 mice in both infected groups compared to their respective control. We also observed a reduction in the MFI of MHCII in IL-17RA^-/-^ 3dpi mice compared to IL-17RA^-/-^ 0dpi (**Figures 7G–J**). In the dimensional reduction analysis, we observed that the E1 cluster is present in all groups except for WT 0dpi, while E2, although present in all groups, stands out in the uninfected groups (**Figures 7P, Q**).

In neutrophils, we observed a reduction in MHCII and IL-10 in the infected groups compared to their respective control groups. We also observed an increase in IL-4 in these cells in IL-17RA^-/-^ 3dpi mice compared to WT 3dpi (p = 0.0014). When analyzing the neutrophil populations by dimensional reduction, we observed that the population of N1 neutrophils is found in greater quantity in the infected groups, and they express IL-17 and IL-4, while the N2 population is found in the uninfected groups, and these express IL-10 and MHCII (**Figures 7K–R**).

Thus, we observed that *T. canis* infection induces an increase in IL-17-producing CD4+ T lymphocytes and IL-4 and IL-10-producing eosinophils, and the absence of IL-17RA increases the frequency of IL-4-producing neutrophils and reduces the frequency of alveolar macrophages expressing the same cytokine.

### IL-17RA receptor signaling contributes to the formation of lung inflammatory nodular aggregates during the *T. canis* infection

**Figure 8:**
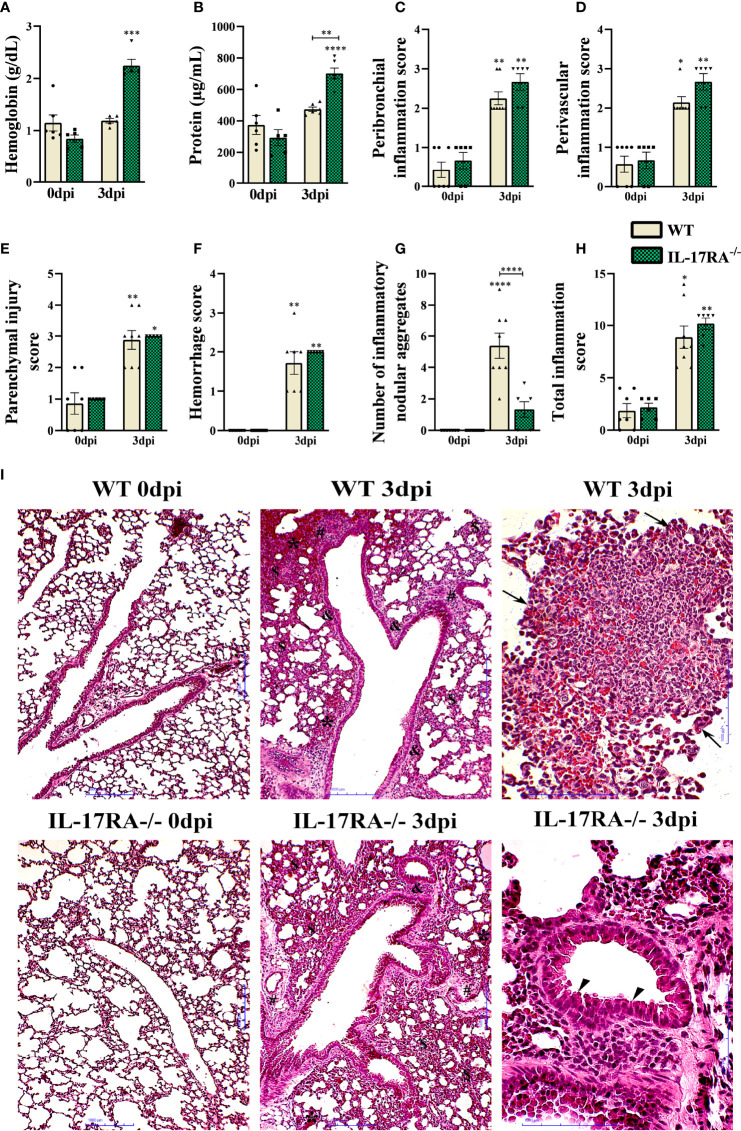
Characterization of lung parenchyma injury and inflammation in WT and IL-17RA-/- mice during T. canis infection. **(A)** Hemoglobin quantification in the bronchoalveolar lavage (BAL); **(B)** Total protein levels in BAL; **(C)** Peribronchial inflammation score; **(D)** Score for perivascular inflammation; **(E)** Parenchymal injury score; **(F)** Bleeding score; **(G)** Number of inflammatory nodular aggregate; **(H)** Total inflammation score; **(I)** Histopathology of lung tissue. Representative hematoxylin and eosin staining of lung sections. Bronchial epithelial hypertrophy and hyperplasia (arrowhead), inflammatory nodular aggregate (arrows), bleeding area (*), parenchymal inflammation ($), airway inflammation (&), vascular inflammation (#). Bar = 1000μm. Statistical analyses were performed between each strain with its uninfected group (0dpi), represented by the asterisk without the bar, and between the two strains at the same time of infection, represented by the asterisk with the bar. Non-significant differences were not reported in the bar. Data represented as mean ± SEM, *p < 0.05, **p < 0.01, ***p < 0.001, ****p < 0.0001. For data that passed the normality test, B, One-way ANOVA test followed by the Tukey test was used, while non-parametric data **(A, C, D, E–H)** were used Kruskal-Wallis test followed by the Dunn test. N = 7 mice per experimental group.

When analyzing the protein and hemoglobin levels in the BAL, the IL-17RA^-/-^ 3dpi mice had a higher protein concentration than the WT 3dpi (p = 0.0057), probably due to the higher parasite load and higher levels of higher cytokine-producing cells regulators (**Figures 8A, B**). In the histopathological analysis, we did not observe significant differences in the inflammation and hemorrhage scores; however, the IL-17RA^-/-^ 3dpi mice reduced the number of inflammatory nodular aggregates (p <0.0001) compared to WT mice 3dpi (**Figures 8C–H**). We also analyzed the activity of N-acetylglucosaminidase (NAG), eosinophilic peroxidase (EPO) and neutrophil myeloperoxidase (MPO) in lung tissue, with no significant difference between the infected groups (**Supplementary Figure 5**).

The histopathological analysis of the lung parenchyma of mice belonging to the group’s WT 0dpi, IL-17RA^-/-^ 0dpi, WT 3dpi, IL-17RA^-/-^ 3dpi infected with *T. canis* was performed. It was possible to observe and describe the lesions of the lung parenchyma caused by larval migration of the parasite in terms of topography, inflammatory infiltrate, presence or absence of larvae, inflammatory nodular aggregates, vascular and exudative phenomena (**Figure 8I**).

Regarding mice belonging to the WT 3dpi group, when the histopathological analysis was performed, thickening of the interalveolar septa was observed at the expense of the mixed inflammatory infiltrate characterized by eosinophils and neutrophils, macrophages and, in a smaller amount, constituted by lymphocytes. Multifocal nodular aggregates constituted predominantly by eosinophils and to a lesser extent by neutrophils, macrophages, and lymphocytes were frequently observed in all mice, often located around larval fragments. Exudative phenomena such as perivascular edema and hemorrhagic areas were also evidenced. Hypertrophy of the epithelial cells of the bronchi and bronchioles has often been observed. Most mice had larvae dispersed in the lung parenchyma and often close to the hemorrhagic zones.

When evaluating the lung parenchyma of mice belonging to the IL-17RA^-/-^ 3dpi group, an increase in the thickening of the interalveolar septa was observed at the expense of the predominantly mononuclear inflammatory infiltrate characterized by macrophages and lymphocytes, and sometimes neutrophils and eosinophils were found. Sometimes, in some mice, the presence of multifocal nodular aggregates constituted predominantly by eosinophils and, to a lesser extent, by neutrophils, macrophages, and lymphocytes were observed. The observed nodular aggregates were smaller and smaller when compared to those observed in the WT 3dpi group. Exudative phenomena such as perivascular edema, large hemorrhagic areas, and vascular congestion were frequently evidenced, contributing to the thick appearance of the alveolar septa. Hypertrophy and hyperplasia of the bronchial epithelial cells were also frequently observed. In all mice, larvae were found scattered in the lung parenchyma and often close to the hemorrhagic zones. The histopathological analysis of the lung parenchyma of mice belonging to the control groups WT 0dpi, IL-17RA^-/-^ 0dpi exhibited a regular morphological aspect, without histopathological alterations.

## Discussion

In toxocariasis, the immune response triggered during infection is associated with eosinophilia in peripheral blood, eosinophilic infiltration around larval migration sites, and a production of type 2 (Th2) T helper immune response (20). Recent studies demonstrate that several cytokines are present during the acute and chronic phases of the disease, and among them is IL-17, which is increased during infection (13, 14). In this way, the study of the role of the IL-17 pathway in toxocariasis becomes essential to expand the immunological knowledge about the disease and provide avenues for the development of drugs and therapeutic targets that can prevent larval migration in accidental hosts. For the study of IL-17A/IL-17RA axis, mice genetically deficient for IL-17RA generated from C57BL/6 mice were used (21).

Upon penetrating the intestinal mucosa in incidental hosts, *T. canis* larvae migrate mainly towards the liver, lungs, and brain. They may also travel to other organs (e.g., skeletal muscle, heart, and eyes) by mechanical means and digestion by protease (16). The presence of the larva triggers an acute inflammatory reaction, resulting mainly from innate immunity to *T. canis* excretion-secretion (TES) antigens (22). In our study, we observed an increase in the parasite load in the lungs of IL-17RA mice at 3dpi. In a previous study, a change in larval load was also observed with 3dpi in the lungs in GATA1^-/-^ mice infected with *T. canis* compared to WT (23). Although parasite migration is erratic, at 3dpi is the peak of larval migration in the lung (13), thus we found a greater number of larvae at this time of infection, which may have shown greater statistical differences between the groups.

Although there is scarce data related to IL-17/IL-17RA axis in toxocariasis in the literature, studies relating IL-17 to helminth infections have expanded. Nogueira et al. (9) observed that mice, after multiple exposures to *Ascaris suum*, exhibited greater control of larval migration due to intense lung inflammation associated with a systemic Th2/Th17 immune response. In murine schistosomiasis, the absence of IL-17A signaling has been related to reduced liver fibrosis (6). Resende et al. (13) demonstrated that in toxocariasis, there is a mixture of Th2 and Th17 inflammatory responses, observed by the increase of the cytokines IL-4, IL-5, IL-13, IL-6, and IL-17 in the serum of mice, during the phase of larval migration, showing that *T. canis* larvae are capable of triggering IL-17 production. Previous studies by our group also observed that the IL-33/ST2 pathway in mice infected with *T. canis* increased hepatic and brain parasite load and reduced IL-17 activity (14). In our study, the presence of IL-17RA appears to be relevant to reduce the parasite load in the acute phase of infection in the lungs, and this control in the initial phase is essential to prevent a greater number of larvae from migrating to more susceptible organs, such as the brain.

In *T. canis* infection, leukocytosis is expected, with neutrophil recruitment followed by eosinophil activation, which is usually increased during the acute inflammation phase but may persist until the chronic phase of infection (24). In our study, leukocytosis was observed only in IL-17RA^-/-^ 3dpi mice. The cytokine IL-17 is important for neutrophil biology, also is a potent stimulator of lung microvascular endothelial cells to produce the neutrophil chemoattractant (CXCL8 and derivatives of the 5-lipoxygenase pathway) that selectively recruits these cells to sites of inflammation (25). Therefore, the absence of leukocytosis in WT mice is due to the targeting of immune cells to the tissues, especially the lungs, as shown in our study.

Classical monocytes are essential for initiating the inflammatory response, while non-classical monocytes have been widely viewed as an anti-inflammatory, maintaining vascular homeostasis. When analyzing the cells present in the blood of WT and IL-17RA^-/-^ mice, we observed the presence of classic and non-classical monocytes, and in the mice infected by *T. canis*, these cells showed higher expression of TNF-α. TNF-α appears to play a crucial role in regulating the survival and function of monocytes in the periphery (26, 27). In the serum of patients infected with *T. canis*, an increase in TNF-α and IL-10 was observed (28). On the other hand, Resende et al. (13) did not observe significant changes in the levels of IL-10, TNF-α, or IFN-γ in mice infected with *T. canis*. Also, in the peripheral blood of mice infected with *T. canis*, an increase in the cytokines IL-4, IL-5 and IL-10 was observed, but not in TNF-α (20).

As we depicted by the analysis of blood eosinophils using the flow cytometry, the IL-17RA^-/-^ mice showed increased frequencies of eosinophils during *T. canis* infection compared to WT mice. In response to damage or some types of infections, eosinophils are recruited to sites of inflammation where they secrete cytokines such as IL-2, IL-4, IL-5, IL-10, IL-12, IL-13, IL- 16, IL-18, TGF, toxic granular proteins, lipid mediators, in addition to being capable of inducing tissue damage and dysfunction (29). It has also been shown that eosinophils constitutively express receptors for IL-17A and IL-17F, and it is hypothesized that their Th17-mediated activation could induce the release of pro-inflammatory cytokines and chemokines (30).

Although classically described the importance of CD4+ and CD8+ T lymphocytes, γδ T lymphocytes and NK cells in the expression of IL-17, studies have shown that eosinophils and neutrophils can produce large amounts of IL-17 in the lung, which is related to inflammation lung disease in allergic models and in infections (2, 31-36). Moreover, eosinophil-derived IL-17 contributes to lung tissue injury and inflammation, controlling the expansion of IL-17+ Th17 and Tγδ lymphocytes during *Aspergillus fumigatus* infection in mice (2). Thus, in the absence of the IL-17A receptor, eosinophils seem to be recruited to amplify the IL-17A response and, consequently, tissue inflammation. Neutrophils are the first cells of innate immunity to reach the injury site. In the present study, they were shown to be producers of IL-17 and IL-4, mainly in infected mice. Studies have reported the role of neutrophils as IL-17 producers in psoriasis, autoimmune diseases, bacterial infections, and helminth infections (37–39). For example, in Nippostrongylus brasiliensis infection, an alternatively activated (N2) neutrophil phenotype leads to developing a long-lasting M2 macrophage phenotype, which subsequently mediates parasitic larval damage (40). Rodolpho et al. (41) demonstrated that in BALB/c mice during *T. canis* infection, peripheral blood eosinophils show an upregulated expression of activation markers such as CD69, MHCII, CD80, CD86, as demonstrated in our study.

CD27 is a co-stimulation molecule expressed on naive CD4+ and TCD8+ T lymphocytes and, unlike other members of the TNF receptor family, is released from the cell surface after T cell activation (42). CD69 is a marker expressed on the surface of T lymphocytes after the involvement of the T cell receptor (TCR) with CD3. This marker activates cytokines, performs mitogenic polyclonal stimulation, carries out the targeting and migration of lymphocytes, and appears to be an early controller of Th17 differentiation, preventing the differentiation of T cells towards Th17 (43, 44). In our study, we did not observe differences in the activation markers CD27, MHCII and CD69. Our results were probably due to the evaluation of the infection time, which was at 3dpi, not being sufficient to assess the differentiation and activation of lymphocytes.

During migration, *T. canis* larvae cause tissue damage and provoke inflammatory reactions, with the involvement of neutrophils, eosinophils, and lymphocytes in the infiltrate of the lungs of infected mice (45). Our study observed an increase in IL-6 cytokine in IL-17RA^-/-^ mice compared to WT. The IL-17RA receptor is expressed in the lungs on lung fibroblasts, lung endothelial cells, and airway smooth muscle cells; signaling through this receptor induces the production of chemokines and the cytokine IL-6 (46).

In our study, we observed a reduction in the percentage of neutrophils and an increase in macrophages in the BAL of IL-17RA^-/-^ infected mice. The IL-17 pathway is essential in the recruitment and activation of macrophages and especially neutrophils to fight extracellular bacteria and fungi on mucosal surfaces. Contributes to epithelial homeostasis in the skin and stimulates B cells, acting as a bridge between the innate and acquired immune system (36). Our data also showed that IL-4 and IL-17 seem to be important in the response to *T. canis* infection, produced by various BAL cells, such as macrophages, eosinophils and mainly by neutrophils, as demonstrated by the cytometry technique. Neutrophils express and produce cytokines constitutively or upon activation by microenvironmental stimulus and can be a significant source of pro-inflammatory and immunoregulatory cytokines. The main neutrophil secreted cytokines are IL-17 and IFN-γ; however, studies have also reported the production of IL-4, IL-10, and TGF-β (47). Our study indicated that neutrophils that express IL-17 are more present in mice infected with *T. canis*, suggesting that the microenvironment generated by the infection favors the production of this cytokine.

Alveolar macrophages are the most abundant innate immune cells in the distal lung, located on the luminal surface of the alveolar space and due to exposure to the high partial pressure of oxygen, surfactant, and signals provided by alveolar type I and II cells displayed a phenotype distinct from other cells, which allows them to be differentiated from transient monocyte-derived cells recruited into the alveolar space during tissue injury. These macrophages are identified by the expression of high levels of CD11c integrin and the lectin Siglec F (48). M2 macrophages are primarily activated by IL-4/IL-13 and IL-10 in response to injury, and they act to promote wound healing by attenuating inflammation and stimulating extracellular matrix formation (49, 50). Studies have shown that depending on the stimulus and if there are macrophages, they can secrete Th2 cytokines such as IL-4 and IL-13 (51, 52). Opsonized schistosome eggs antigens (SEA) have been shown to upregulate IL-4 production by C57BL/6 macrophages (52), as demonstrated in our study, in which there was an increase in the percentage of IL-4 in macrophages in WT 3dpi mice. In our study, we observed that the absence of the IL-17A receptor caused these macrophages to decrease the production of IL-4, we believe that the absence of IL-17RA changes the cytokine microenvironment to which macrophages are exposed, and in this way may even interfere on the type of differentiation in classical or alternatively activated macrophages.

In our study, we observed that several cells are sources of IL-17A and although the effect of these cytokines is more related to the induction of inflammation, IL-17 is not as potent in isolation. Its primary function is to recruit immune cells and act synergistically with other cytokines such as TNF, IL-1β, IFN-γ, GM-CSF and IL-22 (5, 53). Studies have indicated that there is a close link between IL-17A and the Th2 response, and it has been observed that in the initial phase of infection by *N. brasiliensis* there is a reduction in the levels of IFN-γ mediated by IL-17A, allowing the subsequent development of immunity type 2 in the lungs (12). However, while there is much evidence that effector cytokines on the IL-17/IL-17RA axis may play protective roles against infectious agents in the lung, there is increasing evidence that this pathway can also result in lung pathology (46). Thus, we believe that in the initial phase of infection by *T. canis*, the cytokines IL-17A and IL-4 are the main active components, however, a synergistic action with other components of the immune system is necessary for a more effective and controlled response.

Pulmonary infections by *T. canis* are characterized by pulmonary inflammation with cell aggregates, the presence of hemoglobin and protein extravasation, which alter vascular permeability, airway hyperresponsiveness, and, frequently, the presence of granulomas are observed (14, 45, 54, 55). In a previous study, BALB/c mice infected with *T. canis* with 3dpi showed thickening of the interalveolar septa with a mixed inflammatory infiltrate in the lungs, characterized by eosinophils, neutrophils, macrophages, and lymphocytes. The mice presented exudative phenomena, such as perivascular edema and extensive hemorrhagic areas, in addition to the presence of granulomas in the exudative phase, composed mainly of eosinophils and macrophages followed by lymphocytes (14). The infected mice in our study showed similar results; however, the IL-17RA^-/-^ 3dpi mice showed an increase in hemoglobin and inflammatory nodular aggregates formed mainly by eosinophils, which later gave rise to granulomas. IL-17A plays a significant role in granuloma maturation, from early to mature stages, and is indispensable for the protective response against *Mycobacterium tuberculosis* infection in the lung (56). Paracoccidioidomycosis also showed that IL-6, IL-23, or IL-17RA receptor deficiency impaired granuloma formation and conferred susceptibility during infection (57). Thus, we demonstrate that the presence of the IL-17RA receptor is essential for the formation of inflammatory nodular aggregates, amplifying the inflammatory process that can generate greater tissue damage; however, as it is in the initial phase of infection, lung damage was not observed in these mice.

**Figure 9:**
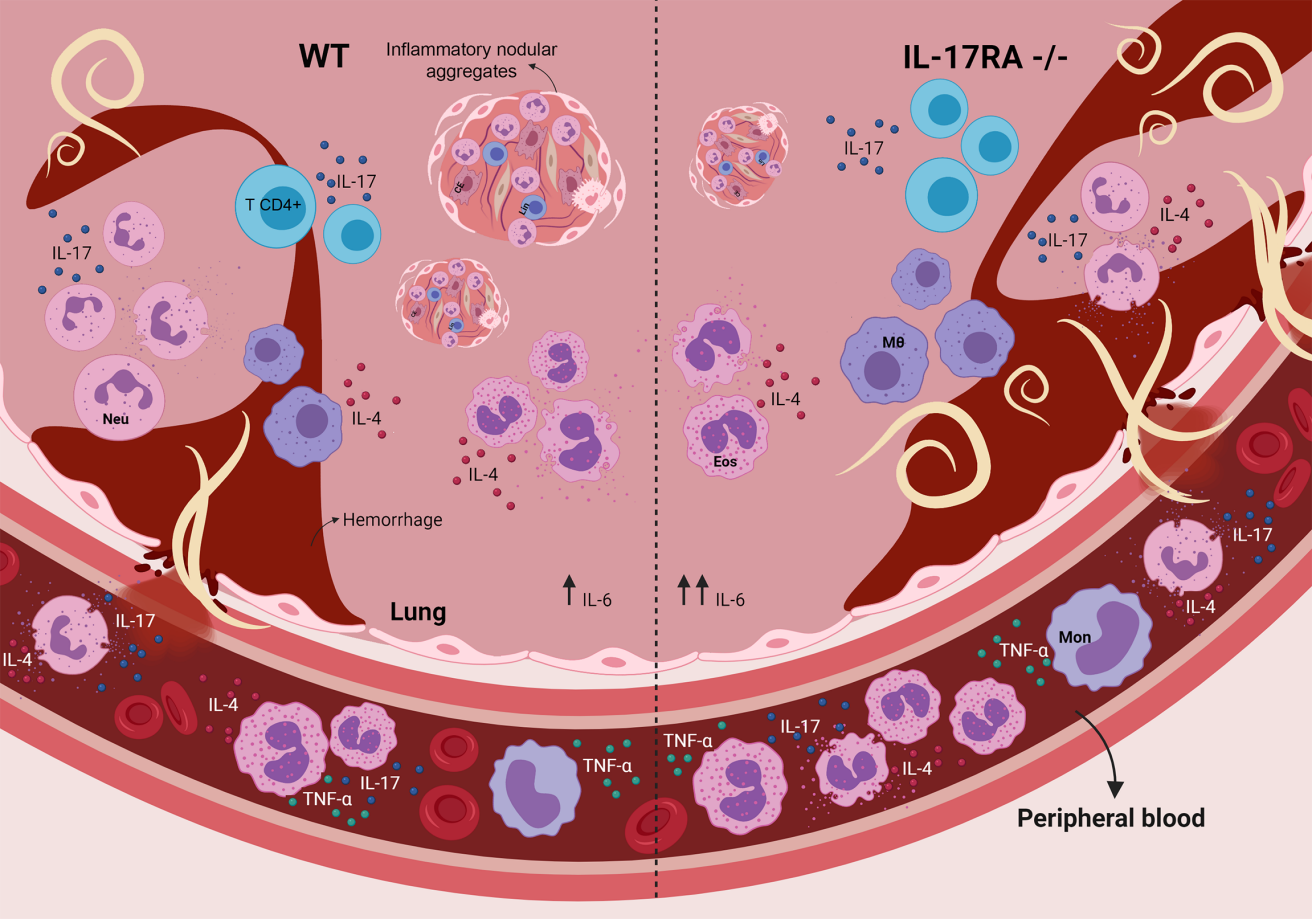
Role of the IL-17RA receptor in the context of T. canis infection in mice. The absence of the IL-17RA receptor during T. canis infection increases the frequency of eosinophils in the blood, contributes to the increase in the pulmonary parasite load, IL-6 production and the frequency of IL-4-secreting neutrophils and reduces the number of inflammatory nodular aggregators. MΦ, alveolar macrophage; CE, endothelial cell; Eos, eosinophil; Neu, neutrophil; Mon, monocyte; Lin, lymphocyte.

Taken together, this study demonstrates that the IL-17/IL-17RA axis contributes to reducing parasite burden but increases tissue inflammation during the acute phase of *T. canis* infection. Furthermore, the production of the cytokines IL-17A and IL-4, originating mainly from innate immune cells, seems to be important for controlling the larval lung burden at the beginning of the infection (**Figure 9**). However, the IL-17RA pathway triggers the formation of pulmonary nodular aggregates that suggest a future formation of granulomas that amplify the lung injury. In conclusion, our results suggest that, in the context of toxocariasis, the IL-17RA receptor may represent a promising therapeutic target to reduce organ inflammation and morbidity triggered by uncontrolled parasite migration, causing neurotoxocariasis and ocular toxocariasis. Finally, future studies are needed to reveal the mechanisms of pharmacological modulation of IL-17A during the parasite-host relationship in the context of toxocariasis.

## Data availability statement

The original contributions presented in the study are included in the article/**Supplementary Material**. Further inquiries can be directed to the corresponding author.

## Ethics statement

The maintenance and use of mice were carried out following the recommendations of the Brazilian College of Animal Experimentation (COBEA). The present study was reviewed and approved by the Ethics Committee for Animal Experimentation (CEUA) of the Federal University of Minas Gerais, Brazil, through protocol #56/2018. All efforts were made to minimize animal suffering.

## Author contributions

Conceived and designed the experiments: TL-S, CL, FV-S, FO, RF, LM, LB. Performed the experiments: TL-S, CL, FV-S, FO, LK, LP, CA, JS, LM. Analyzed the data: TL-S, FO, RR, LM, LB. Contributed reagents, materials, analysis tools: TL-S, FO, RR, RF, LM, LB. Wrote and reviewed the paper: TL-S, CL, FV-S, FO, LK, LP, CA, JN, RR, RF, LM, LB. All authors contributed to the article and approved the submitted version.

## Funding

This investigation received partial support from Fundação de Amparo a Pesquisa do Estado de Minas Gerais/FAPEMIG, Brazil (Grant# CBB APQ-00766-18), the Brazilian National Research Council (CNPq) (Grant# 421392/2018-5 and Grant# 302491/2017-1) and Pró-Reitoria de Pesquisa of Universidade Federal de Minas Gerais to cover research inputs. TL-S is grateful for the Ph.D. fellowship provided by the Brazilian National Research Council (CNPq), Post-graduation Program in Infectology and Tropical Medicine/Universidade Federal de Minas Gerais. RR, RF, and LB are Research Fellows from the Brazilian National Research Council (CNPq). The funders had no role in study design, data collection, analysis, decision to publish, publication fees, or manuscript preparation.

## Conflict of interest

The authors declare that the research was conducted in the absence of any commercial or financial relationships that could be construed as a potential conflict of interest.

The handling editor declared a past co-authorship with the authors TL-S, RF, LM, and LB.

## Publisher’s note

All claims expressed in this article are solely those of the authors and do not necessarily represent those of their affiliated organizations, or those of the publisher, the editors and the reviewers. Any product that may be evaluated in this article, or claim that may be made by its manufacturer, is not guaranteed or endorsed by the publisher.

## References

[1] O’Brien RL Roark CL Born WK. Il-17-producing γδ t cells. Eur J Immunol (2009) 39:662–6. doi: 10.1002/eji.200839120 PMC2698711 19283718

[2] de Oliveira Malacco NLS Rachid MA Gurgel ILDS Moura TR Sucupira PHF de Sousa LP. Eosinophil-associated innate IL-17 response promotes aspergillus fumigatus lung pathology. Front Cell Infect Microbiol (2019) 9:453. doi: 10.3389/fcimb.2018.00453 30687649 PMC6336733

[3] Kim HY Lee HJ Chang YJ Pichavant M Shore SA Fitzgerald KA. Interleukin-17-producing innate lymphoid cells and the NLRP3 inflammasome facilitate obesity-associated airway hyperreactivity. Nat Med (2014) 20:54–61. doi: 10.1038/nm.3423 24336249 PMC3912313

[4] Gaffen SL. Structure and signaling in the IL-17 receptor superfamily. Nat Rev Immunol (2010) 9:1–24. doi: 10.1038/nri2586.Structure PMC2821718 19575028

[5] Ruiz de Morales JMG Puig L Daudén E Cañete JD Pablos JL Martín AO. Critical role of interleukin (IL)-17 in inflammatory and immune disorders: An updated review of the evidence focusing in controversies. Autoimmun Rev (2020) 19:102429. doi: 10.1016/j.autrev.2019.102429 31734402

[6] Zhang Y Huang D Gao W Yan J Zhou W Hou X. Lack of Il-17 signaling decreases liver fibrosis in murine schistosomiasis japonica. Int Immunol (2015) 27:317–25. doi: 10.1093/intimm/dxv017 25840437

[7] Rutitzky LI Stadecker MJ. Exacerbated egg-induced immunopathology in murine schistosoma mansoni infection is primarily mediated by IL-17 and restrained by IFN-γ. Eur J Immunol (2011) 41:2677–87. doi: 10.1002/eji.201041327 PMC3679923 21660933

[8] Chen D Xie H Luo X Yu X Fu X Gu H. Roles of Th17 cells in pulmonary granulomas induced by schistosoma japonicum in C57Bl/6 mice. Cell Immunol (2013) 285:149–57. doi: 10.1016/j.cellimm.2013.09.008 24212062

[9] Nogueira DS Gazzinelli-Guimarães PH Barbosa FS Resende NM Silva CC de Oliveira LM. Multiple exposures to ascaris suum induce tissue injury and mixed Th2/Th17 immune response in mice. PloS Negl Trop Dis (2016) 10:1–19. doi: 10.1371/journal.pntd.0004382 PMC4729520 26814713

[10] Sutherland TE Logan N Rückerl D Humbles AA Allan SM Papayannopoulos V. Chitinase-like proteins promote Il-17-mediated neutrophilia in a tradeoff between nematode killing and host damage. Nat Immunol (2014) 15:1116–25. doi: 10.1038/ni.3023 PMC4338525 25326751

[11] Chen F Liu Z Wu W Rozo C Bowdridge S Millman A. An essential role for T H 2-type responses in limiting acute tissue damage during experimental helminth infection. Nat Med (2012) 18:260–6. doi: 10.1038/nm.2628 PMC3274634 22245779

[12] Ajendra J Chenery AL Parkinson JE Chan BHK Pearson S Colombo SAP. Il-17a both initiates, *via* IFNγ suppression, and limits the pulmonary type-2 immune response to nematode infection. Mucosal Immunol (2020) 13:958–68. doi: 10.1038/s41385-020-0318-2 PMC7567645 32636457

[13] Resende NM Gazzinelli-Guimarães PH Barbosa FS Oliveira LM Nogueira DS Gazzinelli-Guimarães AC. New insights into the immunopathology of early toxocara canis infection in mice. Parasites Vectors (2015) 8:354. doi: 10.1186/s13071-015-0962-7 26135397 PMC4494798

[14] Leal-Silva T Vieira-Santos F Oliveira FMS Padrão LLS Kraemer L da Paixão Matias PH. Detrimental role of IL-33/ST2 pathway sustaining a chronic eosinophil-dependent Th2 inflammatory response, tissue damage and parasite burden during toxocara canis infection in mice. PloS Negl Trop Dis (2021) 15:e0009639. doi: 10.1371/journal.pntd.0009639 34324507 PMC8354467

[15] Rostami A Riahi SM Holland CV Taghipour A Khalili-Fomeshi M Fakhri Y. seroprevalence estimates for toxocariasis in people worldwide: A systematic review and meta-analysis. PloS Negl Trop Dis (2019) 13:e0007809. doi: 10.1371/journal.pntd.0007809 31856156 PMC6922318

[16] Ma G Holland CV Wang T Hofmann A Fan CK Maizels RM. Human toxocariasis. Lancet Infect Dis (2018) 18:e14–24. doi: 10.1016/S1473-3099(17)30331-6 28781085

[17] Pecinali NR Gomes RN Amendoeira FC Bastos AC Martins MJ Pegado CS. Influence of murine toxocara canis infection on plasma and bronchoalveolar lavage fluid eosinophil numbers and its correlation with cytokine levels. Vet. Parasitol (2005) 134:121–30. doi: 10.1016/j.vetpar.2005.06.022 16168564

[18] Novák J Panská L Machá č ek T Kolá ř ová L Horák P. Humoral response of mice infected with toxocara canis following different infection schemes. Acta Parasitol (2017) 62:823–35. doi: 10.1515/ap-2017-0099 29035857

[19] Chen H Lau MC Wong MT Newell EW Poidinger M Chen J. Cytofkit: a bioconductor package for an integrated mass cytometry data analysis pipeline. PloS Comput Biol (2016) 12:1–17. doi: 10.1371/journal.pcbi.1005112 PMC5035035 27662185

[20] Ruiz-Manzano RA Hernández-Cervantes R del Río-Araiza VH Palacios-Arreola MI Nava-Castro KE Morales-Montor J. Immune response to chronic toxocara canis infection in a mice model. Parasite Immunol (2019) 41:1–11. doi: 10.1111/pim.12672 31557337

[21] Floudas A Saunders SP Moran T Schwartz C Hams E Fitzgerald DC. IL-17 receptor a maintains and protects the skin barrier to prevent allergic skin inflammation. J Immunol (2017) 199:707–17. doi: 10.4049/jimmunol.1602185 PMC5509014 28615416

[22] Jaramillo-Hernández DA Salazar-Garcés LF Baquero-Parra MM da Silva-Pinheiro C Alcantara-Neves NM. Toxocariasis y vacunación para toxocara: una revisión sistemática. Orinoquia (2020) 24:79–95. doi: 10.22579/20112629.631

[23] Leal-Silva T Lopes C de A Vieira-Santos F Oliveira FMS Kraemer L Padrão L de LS. Tissue eosinophilia correlates with mice susceptibility, granuloma formation, and damage during toxocara canis infection. Parasitology (2022), 149 (7) 1–38. doi: 10.1017/s0031182022000075 35139931

[24] Holland CV Smith HV. eds. Toxocara: the enigmatic parasite. Oxfordshire, UK Publishing C (2006).

[25] Roussel L Houle F Chan C Yao Y Bérubé J Olivenstein R. IL-17 Promotes p38 MAPK-dependent endothelial activation enhancing neutrophil recruitment to sites of inflammation. J Immunol (2010) 184:4531–7. doi: 10.4049/jimmunol.0903162 20228195

[26] Babu Narasimhan P Marcovecchio P Hamers AA Hedrick CC. Nonclassical Monocytes in Health and Disease. Annual Rev Immunol (2019) 37:439-56. doi: 10.1146/annurev-immunol-042617 31026415

[27] Wol Y Shemer A Polonsky M Gross M Mildner A Yona S. Autonomous TNF is critical for *in vivo* monocyte survival in steady state and inflammation. J Exp Med (2017) 214:905–17. doi: 10.1084/jem.20160499 PMC5379969 28330904

[28] Olaniyan M Azeez M. Seroprevalence of toxocara canis and the parasitic effect on plasma cytokines in children aged 6 to 11 years in saki-east local government area in nigeria. Environ Dis (2019) 4:12. doi: 10.4103/ed.ed_23_18

[29] Rothenberg ME Hogan SP. The eosinophil. Annu Rev Immunol (2006) 24:147–74. doi: 10.1146/annurev.immunol.24.021605.090720 16551246

[30] Cheung PFY Wong CK Lam CWK. Molecular mechanisms of cytokine and chemokine release from eosinophils activated by IL-17A, IL-17F, and IL-23: implication for Th17 lymphocytes-mediated allergic inflammation. J Immunol (2008) 180:5625–35. doi: 10.4049/jimmunol.180.8.5625 18390747

[31] Lambrecht BN Hammad H. The immunology of asthma. Nat Immunol (2015) 16:45–56. doi: 10.1038/ni.3049 25521684

[32] Taylor PR Roy S Leal SM Sun Y Howell SJ Cobb BA. Activation of neutrophils by autocrine IL-17A-IL-17RC interactions during fungal infection is regulated by IL-6, IL-23, RORγt and dectin-2. Nat Immunol (2014) 15:143–51. doi: 10.1038/ni.2797 PMC3972892 24362892

[33] Molet S Hamid Q Davoine F Nutku E Taha R Pagé N. IL-17 is increased in asthmatic airways and induces human bronchial fibroblasts to produce cytokines. J Allergy Clin Immunol (2001) 108:430–8. doi: 10.1067/mai.2001.117929 11544464

[34] Yadav B Specht CA Lee CK Pokrovskii M Huh JR Littman DR. Lung eosinophils elicited during allergic and acute aspergillosis express RORϪt and IL-23R but do not require IL-23 for IL-17 production. PloS Pathog (2021) 17: e1009891. doi: 10.1371/journal.ppat.1009891 PMC8437264 34464425

[35] Guerra ES Lee CK Specht CA Yadav B Huang H Akalin A. Central role of IL-23 and IL-17 producing eosinophils as immunomodulatory effector cells in acute pulmonary aspergillosis and allergic asthma. PloS Pathog (2017) 13: e1006175. doi: 10.1371/journal.ppat.1006175 PMC5271415 28095479

[36] Fei M Bhatia S Oriss TB Yarlagadda M Khare A Akira S. TNF-α from inflammatory dendritic cells (DCs) regulates lung IL-17A/IL-5 levels and neutrophilia versus eosinophilia during persistent fungal infection. Proc Natl Acad Sci USA (2011) 108:5360–5. doi: 10.1073/pnas.1015476108 PMC3069210 21402950

[37] Hu S He W Du X Yang J Wen Q Zhong XP. IL-17 production of neutrophils enhances antibacteria ability but promotes arthritis development during mycobacterium tuberculosis infection. EBioMedicine (2017) 23:88–99. doi: 10.1016/j.ebiom.2017.08.001 28821374 PMC5605331

[38] Li L Huang L Vergis AL Ye H Bajwa A Narayan V. IL-17 produced by neutrophils regulates IFN-γ-mediated neutrophil migration in mouse kidney ischemia-reperfusion injury. J Clin Invest (2010) 120:331–42. doi: 10.1172/JCI38702 PMC2798679 20038794

[39] Lin AM Rubin CJ Khandpur R Wang JY Riblett M Yalavarthi S. Mast Cells and Neutrophils Release IL-17 through Extracellular Trap Formation in Psoriasis. J Immunol (2011) 187:490–500. doi: 10.4049/jimmunol.1100123 21606249 PMC3119764

[40] Chen F Wu W Millman A Craft J Chen E Patel N. Neutrophils prime a long-lived effector macrophage phenotype that mediates accelerated helminth expulsion. Nat Immunol (2014) 15:938–46. doi: 10.1038/ni.2984 10.1038/ni.2984 PMC4479254 25173346

[41] de Almeida Rodolpho JM Camillo L Araújo MSS Speziali E Coelho-dos-Reis JG Correia R de O. Robust phenotypic activation of eosinophils during experimental toxocara canis infection. Front Immunol (2018) 9:64. doi: 10.3389/fimmu.2018.00064 29445372 PMC5797789

[42] Winkels H Meiler S Lievens D Engel D Spitz C Bürger C. CD27 co-stimulation increases the abundance of regulatory T cells and reduces atherosclerosis in hyperlipidaemic mice. Eur Heart J (2017) 38:3590. doi: 10.1093/eurheartj/ehx517 29045618

[43] Cibrián D Sánchez-Madrid F. CD69: from activation marker to metabolic gatekeeper. Eur J Immunol (2017) 47:946–53. doi: 10.1002/eji.201646837 PMC6485631 28475283

[44] Martín P Gómez M Lamana A Cruz-Adalia A Ramírez-Huesca M Ursa MÁ. CD69 Association with Jak3/Stat5 proteins regulates Th17 cell differentiation. Mol Cell Biol (2010) 30:4877–89. doi: 10.1128/mcb.00456-10 PMC2950549 20696842

[45] Pinelli E Withagen C Fonville M Verlaan A Dormans J van Loveren H. Persistent airway hyper-responsiveness and inflammation in toxocara canis-infected BALB/c mice. Clin Exp Allergy (2005) 35:826–32. doi: 10.1111/j.1365-2222.2005.02250.x 15969676

[46] Weaver CT Elson CO Fouser LA Kolls JK. The Th17 pathway and inflammatory diseases of the intestines, lungs, and skin. Annu Rev Pathol.: Mech Dis (2013) 8:477–512. doi: 10.1146/annurev-pathol-011110-130318 PMC3965671 23157335

[47] Tecchio C Micheletti A Cassatella MA. Neutrophil-derived cytokines : facts beyond expression. Front Immunol (2014) 5:1–7. doi: 10.3389/fimmu.2014.00508 25374568 PMC4204637

[48] Joshi N Walter JM Misharin AV. Alveolar Macrophages. Cell Immunol (2018) 330:86–90. doi: 10.1016/j.cellimm.2018.01.005 29370889

[49] Steen EH Wang X Balaji S Butte MJ Bollyky PL Keswani SG. The role of the anti-inflammatory cytokine interleukin-10 in tissue fibrosis. Adv Wound Care (2020) 9:184–98. doi: 10.1089/wound.2019.1032 PMC7047112 32117582

[50] Wynn TA Barron L. Macrophages: Master regulators of inflammation and fibrosis. Semin Liver Dis (2010) 30:245–57. doi: 10.1055/s-0030-1255354 PMC2924662 20665377

[51] Kang CM Jang AS Ahn MH Shin JA Kim JH Choi YS. Interleukin-25 and interleukin-13 production by alveolar macrophages in response to particles. Am J Respir Cell Mol Biol (2005) 33:290–6. doi: 10.1165/rcmb.2005-0003OC 15961726

[52] la Flamme AC Kharkrang M Stone S Mirmoeini S Chuluundorj D Kyle R. Type II-Activated Murine Macrophages Produce IL-4. PloS One (2012) 7. doi: 10.1371/journal.pone.0046989 PMC3465319 23071691

[53] Onishi RM Gaffen SL. Interleukin-17 and its target genes: Mechanisms of interleukin-17 function in disease. Immunology (2010) 129:311–21. doi: 10.1111/j.1365-2567.2009.03240.x PMC2826676 20409152

[54] Buijs J Lokhorst WH Robinson J Nijkamp FP. Toxocara canis-induced murine puImonary infIammation: analysis of cells and proteins in lung tissue and bronchoalveolar lavage fluid. Parasite Immunol (1994). 16: 1 -9. doi: 10.1111/j.1365-3024.1994.tb00297.x 8152829 10.1111/j.1365-3024.1994.tb00297.x

[55] Kayes SG Jones RE Omholt PE. Pulmonary granuloma formation in murine toxocariasis: transfer of granulomatous hypersensitivity using bronchoalveolar lavage cells. J Parasitol (1988) 74:950–6. doi: 10.2307/3282214 3193334

[56] Okamoto Yoshida Y Umemura M Yahagi A O’Brien RL Ikuta K Kishihara K. Essential role of IL-17A in the formation of a mycobacterial infection-induced granuloma in the lung. J Immunol (2010) 184:4414–22. doi: 10.4049/jimmunol.0903332 20212094

[57] Sales F Tristão M Rocha FA Carlos D Ketelut-carneiro N Oliveira C. Th17-inducing cytokines il-6 and il-23 are crucial for granuloma Formation during experimental Paracoccidioidomycosis. Front Immunol (2017) 8: 949. doi: 10.3389/fimmu.2017.00949 PMC5566564 28871251

