# Supplementary material for: IL-17RA receptor signaling contributes to lung inflammation and parasite burden during Toxocara canis infection in mice
Source: Front Immunol. 2022 Jun 29;13:864632. doi: 10.3389/fimmu.2022.864632 (PMC9277699; doi:10.3389/fimmu.2022.864632)
Supplement: Supplementary file 1 [file DataSheet_1.pdf]

Supplemental Table 1. Histopathological scoring system for mice lung

Score 1
Airways Inflammation Score /6
0 = Lack of inflammatory cells around airways - Absent
1 = Some airways have small numbers of cells - Mild
2 = Some airways have significant inflammation. - Moderate
3 = Majority of airways have some inflammation. - Marked
4 = Majority of airways are significantly inflammed - Severe
5 = All of airways are completely inflammed. – Whole

Score 2
Vascular Inflammation Score /6
0 = Lack of inflammatory cells around vessels. - Absent
1 = Some vessels have small numbers of cells. - Mild
2 = Some vessels have significant inflammation. - Moderate
3 = Majority of vessels have some inflammation. - Marked
4 = Majority of vessels are significantly inflammed - Severe
5 = All of vessels are completely inflammed. - Whole

Score 3
Parenchymal Inflammation (at 10X magnification) Score /6
0 = <1% affected
1 = 1-9% affected
2 = 10-29% affected
3 = 30-49% affected
4 = 50-69% affected
5 = >70% affected

Score 4
Hemorrhage Score /4
0=Absence of hemorrhage
1=Small hemorrhage zones (discreet)
2=Presence of significant hemorrhage zones (moderate)
3=Presence of exuberant hemorrhage zones (severe)

Score 5
Inflammatory nodular aggregates (at 10X magnification)
The number of inflammatory nodular aggregates was counted along the entire length of the slide.
