# Supplementary material for: IL-17RA receptor signaling contributes to lung inflammation and parasite burden during Toxocara canis infection in mice
Source: Front Immunol. 2022 Jun 29;13:864632. doi: 10.3389/fimmu.2022.864632 (PMC9277699; doi:10.3389/fimmu.2022.864632)
Supplement: Supplementary file 2 [file DataSheet_2.pdf]

Supplemental Table 2. Markers used in flow cytometry.

Leukocyte panel in bronchoalveolar lavage (BAL)			
**Markers**	**Fluorochrome**	**Clone**	**Dilution**
CD4	Fitc	H129.19	1:100
IL-4	PE	11B11	1:50
Siglec F	PeTxRed	E50-2440	1:400
CD45	PeCy7	30-F11	1:800
CD11c	APC	N418	1:800
IL-17	APCCy7	TC11-18H10	1:50
Viability	Fixable viability stain 700	-	1:1000
I-A/I-E (MHCII)	BV480	M5/114.15.2	1:50
IL-10	BV421	JES5-16E3	1:50
Ly6G	BV570	1A8	1:200
**Panel of lymphocytes, eosinophils, and neutrophils in peripheral blood**			
**Markers**	**Fluorochrome**	**Clone**	**Dilution**
CD69	Fitc	H1.2F3	1:500
IL-4	PE	11B11	1:50
Siglec F	PeTxRed	E50-2440	1:400
CD4	PeCy5	RM4-5	1:800
CD27	PeCy7	LG3A10	1:200
CD8	APC	53-6.7	1:800
IL-17	APCCy7	TC11-18H10	1:50
Viability	Fixable viability stain 700	-	1:1000
I-A/I-E (MHCII)	BV480	M5/114.15.2	1:50
TNF-α	BV421	MP6-XT22	1:200
Ly6G	BV570	1A8	1:200
**Panel of monocyte in peripheral blood**			
**Markers**	**Fluorochrome**	**Clone**	**Dilution**
I-A/I-E (MHCII)	Fitc	AF6-120.1	1:50
Ly6C	PE	AL-21	1:800
CD11b	PeCy5	M1/70	1:200
TNF-α	BV421	MP6-XT22	1:200
CX3CR1	BV605	SA011F11	1:200
Viability	Fixable viability stain 700	-	1:1000
