# Supplementary material for: IL-17RA receptor signaling contributes to lung inflammation and parasite burden during Toxocara canis infection in mice
Source: Front Immunol. 2022 Jun 29;13:864632. doi: 10.3389/fimmu.2022.864632 (PMC9277699; doi:10.3389/fimmu.2022.864632)
Supplement: Supplementary file 3 [file DataSheet_3.pdf]

# Supplementary Material

## 1 Supplementary Figures

**Supplementary Figure 1 |** Flow cytometry gating strategy. (A) BAL cytometry gating strategy. (A) Analysis strategy. G1: Alveolar macrophages identified by Live, CD45+, SiglecF+ and CD11c+; G2: CD4+ T lymphocytes identified by Live, CD45+, low granularity (SSC) CD4+. G3: Eosinophils identified by Live, CD45+, CD11c- and SiglecF+. G4: Neutrophils identified by Live, CD45+, CD11c-, SiglecF- and Ly6G+. (B) Peripheral blood cytometry gating strategy to identify lymphocytes, eosinophils, and neutrophils. G1: Eosinophils (Live, SiglecF+), G2: Neutrophils (Live, SiglecF-, Ly6G+) G3: CD4+ T lymphocytes (Live,SiglecF-Ly6G-SSC$^{ow}$CD4+), G4: CD8+ T lymphocytes (Live,SiglecF-Ly6G-SSC$^{ow}$CD8+). (C) Gating strategy for identifying monocytes in peripheral blood. G1: Monocytes (Live, CD11b+, CX3CR1+); G2: Classic monocytes (Live, CD11b+, CX3CR1+, Ly6C$^{high}$); G3: Non-classical monocytes (Live, CD11b+, CX3CR1+, Ly6C$^{low}$).

**Supplementary Figure 2 |** (A) Expression level plot from monocyte tSNE depicting expression of CX3CR1, CD11b, Ly6C pattern. (B) tSNE gated in Live CD11b+CX3CR1+ monocyte cells show WT 0dpi mice (red), WT 3dpi mice (green), IL-17RA$^{-/-}$ 0 dpi mice (blue) and IL-17RA$^{-/-}$ 3dpi mice (purple). (C) tSNE gated in Live CD11b+CX3CR1+ monocyte cells show classical monocytes in red (Ly6C$^{high}$) and nonclassical monocytes in blue (Ly6C$^{low}$) of classical (CD11b +, CX3CR1 +, Ly6C$^{high}$) and non-classical (CD11b +, CX3CR1 +, Ly6C$^{low}$) monocytes by experimental group.

**Supplementary Figure 3 |** (A) tSNE gated in Live SiglecF+ eosinophils cells shown in red and SiglecF-Ly6G+ neutrophils cells in blue by experimental group. (B) Expression level plot from eosinophils and neutrophils tSNE depicting expression of SiglecF, Ly6G pattern.

**Supplementary Figure 4 |** Respiratory function analysis of WT and IL-17RA$^{-/-}$ mice not infected and infected by *T. canis*. Variables from lung mechanics were quantified: Forced vital capacity, Chord compliance, Tidal Volume, Dynamic compliance, Forced Expiratory Volume, Lung resistance. Statistical analyzes were performed between each strain with its uninfected group (0dpi), represented by the asterisk without the bar, and between the two strains at the same time of infection, represented by the asterisk with the bar. Data represented as mean ± SEM, * $p < 0.05$, ** $p < 0.01$, *** $p < 0.001$, **** $p < 0.0001$. The One-way ANOVA test followed by the Tukey test was used. The evaluation of pulmonary function was carried out in mice WT and IL17RA$^{-/-}$ as described by [9]. The mice were anesthetized by subcutaneous injection to maintain spontaneous breathing and then were tracheostomized and placed on a body plethysmograph, connected to a computer-controlled ventilator (Forced Pulmonary Maneuver System, Buxco Research Systems, Wilmington, USA). First, pressure-controlled ventilation imposed an average breathing frequency of 160 breaths/min on the animal. Under mechanical respiration, the Dynamic Compliance (Cdyn), the Tidal Volume (TV), and Lung Resistance (Rlung) were determined by Resistance and Compliance (RC) test. The quasi-static pressure-volume maneuver was performed to measure the Chord Compliance (Cchord), which inflates the lungs to a standard pressure of +30 cmH2O and then slowly exhales until a negative pressure of -30 cmH2O is reached. Chord compliance was determined at 10 cmH2O pressure. Fast-flow volume maneuver was performed, and the lungs were inflated to +30 cmH2O and afterward immediately connected to a highly negative pressure to enforce expiration until -30 cmH2O. During this maneuver,

the Forced Vital Capacity (FVC) and Forced Expiratory Volume at 100 milliseconds (FEV100) were recorded. Suboptimal maneuvers were rejected, and for each test in every single mouse, at least three acceptable maneuvers were conducted to obtain a reliable mean for all numeric parameters.

**Supplementary Figure 5 |** (A) N-acetylglucosaminidase (NAG) activity in lung tissue; (B) Eosinophil peroxidase (EPO) activity in lung tissue; (C) Neutrophil myeloperoxidase (MPO) activity in lung tissue. Data represented as mean ± SEM, * $p < 0.05$, ** $p < 0.01$, *** $p < 0.001$, **** $p < 0.0001$. The One-way ANOVA test followed by the Tukey test was used. The activities of NAG, EPO and MPO in pulmonary homogenates were detected according to a previously described method [Nogueira et. al 2016, Barcelos et al. 2005]. After tissue homogenization, the homogenate was centrifuged at 1500g for 10 min at 4 °C, and the resulting pellet was examined to determine NAG, MPO and EPO activities. Absorbance was determined by a Versa-Max ELISA Microplate Reader (Molecular Devices, USA) according to the protocol for each assay, and the results are expressed as the optical density (O.D.).

Nogueira DS, Gazzinelli-Guimarães PH, Barbosa FS, Resende NM, Silva CC, de Oliveira LM, et al. Multiple exposures to Ascaris suum induce tissue injury and mixed Th2/Th17 immune response in mice. PLoS Negl Trop Dis. 2016;10:e0004382.

Barcelos LS, Talvani A, Teixeira AS, Vieira LQ. Impaired inflammatory angiogenesis, but not leukocyte influx, in mice lacking TNFR1. J Leukoc Biol. 2005; 78:352–8.

**Supplementary Figure 6 |** (A) Individual samples of peripheral blood monocyte tSNE from main Figure 3 per experimental group (WT 0dpi, WT 3dpi, IL-17RA-/- 0dpi and IL-17RA-/- 3dpi); (B) Individual samples of peripheral blood tSNE from main figure 3 per experimental group (WT 0dpi, WT 3dpi, IL-17RA-/- 0dpi and IL-17RA-/- 3dpi); (B) Individual samples of peripheral blood neutrophil and eosinophil tSNE from main Figure 4 per experimental group (WT 0dpi, WT 3dpi, IL-17RA-/- 0dpi and IL-17RA-/- 3dpi); (C) Individual samples of bronchoalveolar lavage (BAL) lymphocyte, neutrophil and eosinophil tSNE from main Figure 7 per experimental group (WT 0dpi, WT 3dpi, IL-17RA-/- 0dpi and IL-17RA-/- 3dpi).

**Supplemental Table 1.** Histopathological scoring system for mice lung.

**Supplemental Table 2.** Markers used in flow cytometry.
