# Supplementary figures and images for: IL-17RA receptor signaling contributes to lung inflammation and parasite burden during Toxocara canis infection in mice
Source: Front Immunol. 2022 Jun 29;13:864632. doi: 10.3389/fimmu.2022.864632 (PMC9277699; doi:10.3389/fimmu.2022.864632)

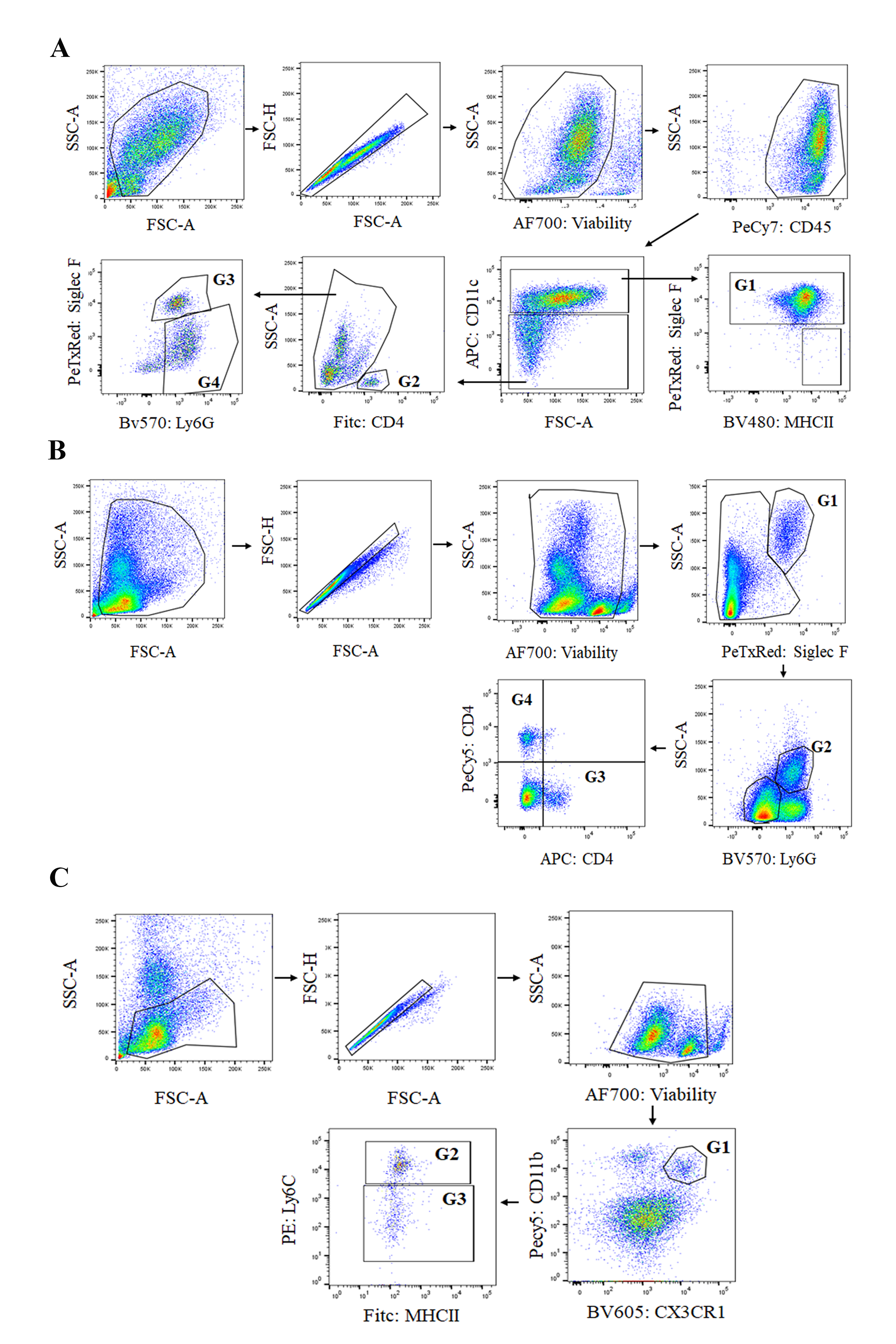

Supplement: Supplementary file 4 [file Image_1.tif]

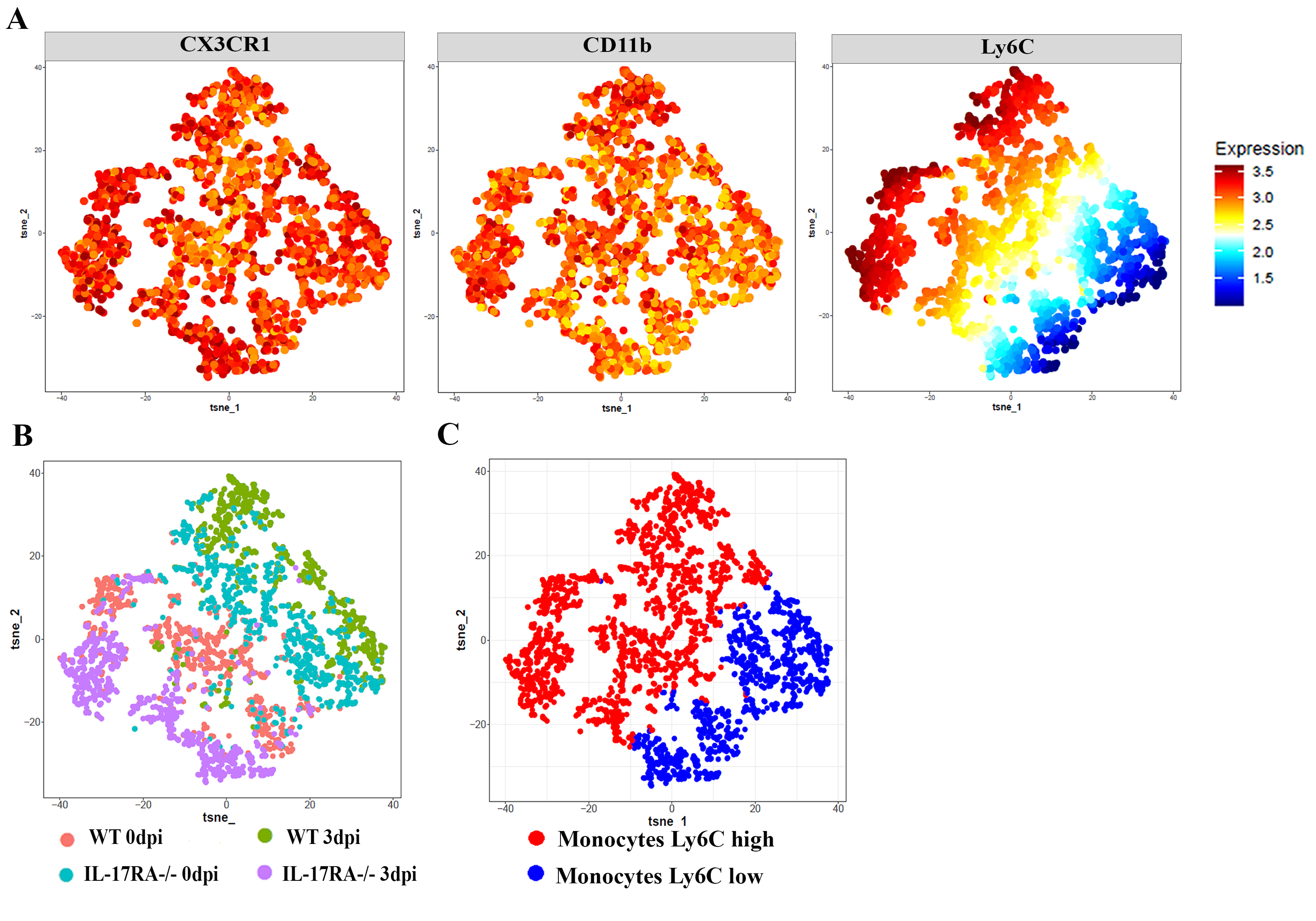

Supplement: Supplementary file 5 [file Image_2.tif]

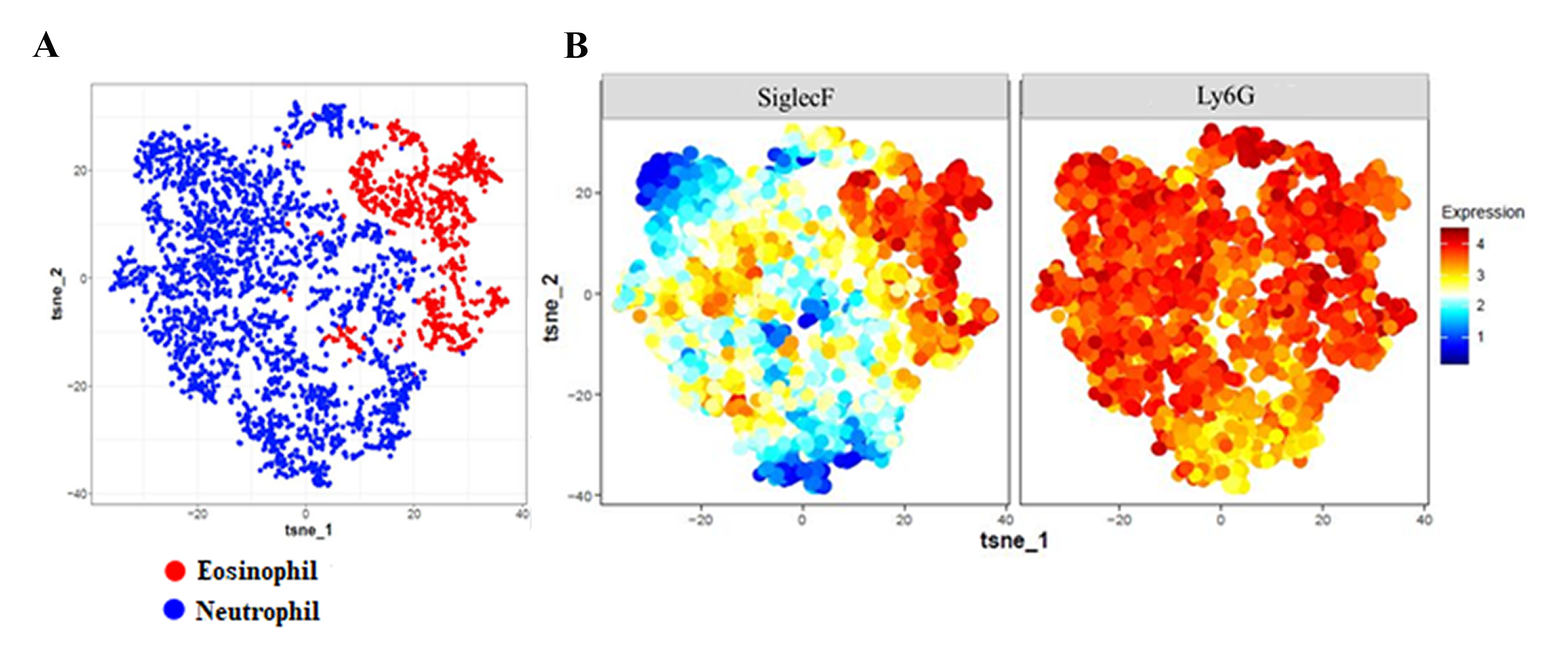

Supplement: Supplementary file 6 [file Image_3.tif]

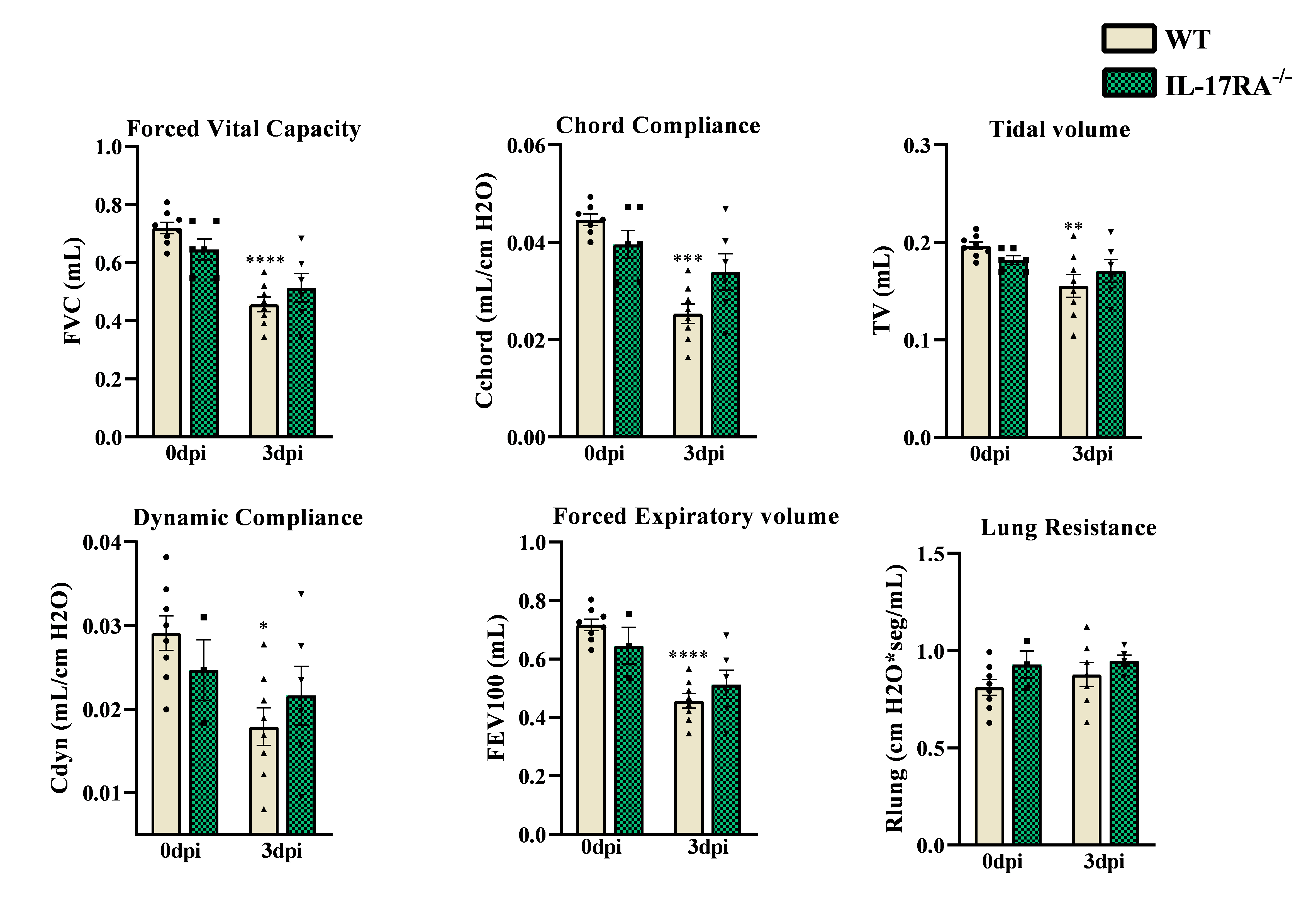

Supplement: Supplementary file 7 [file Image_4.tif]

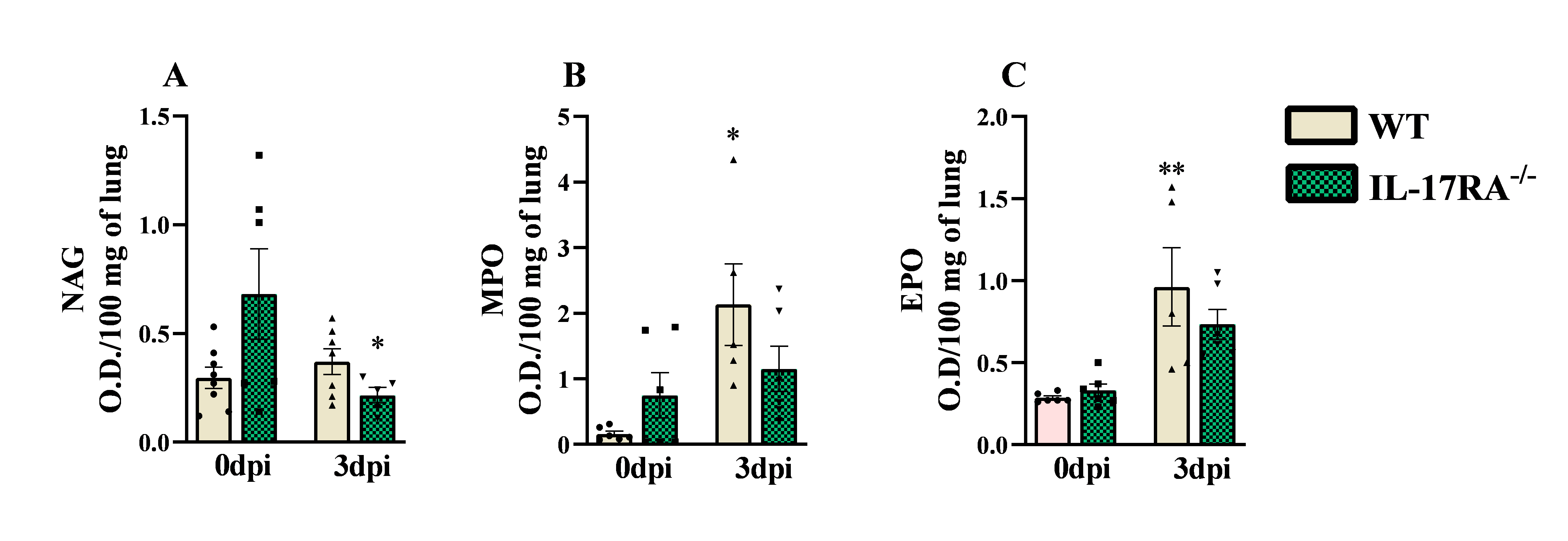

Supplement: Supplementary file 8 [file Image_5.tif]

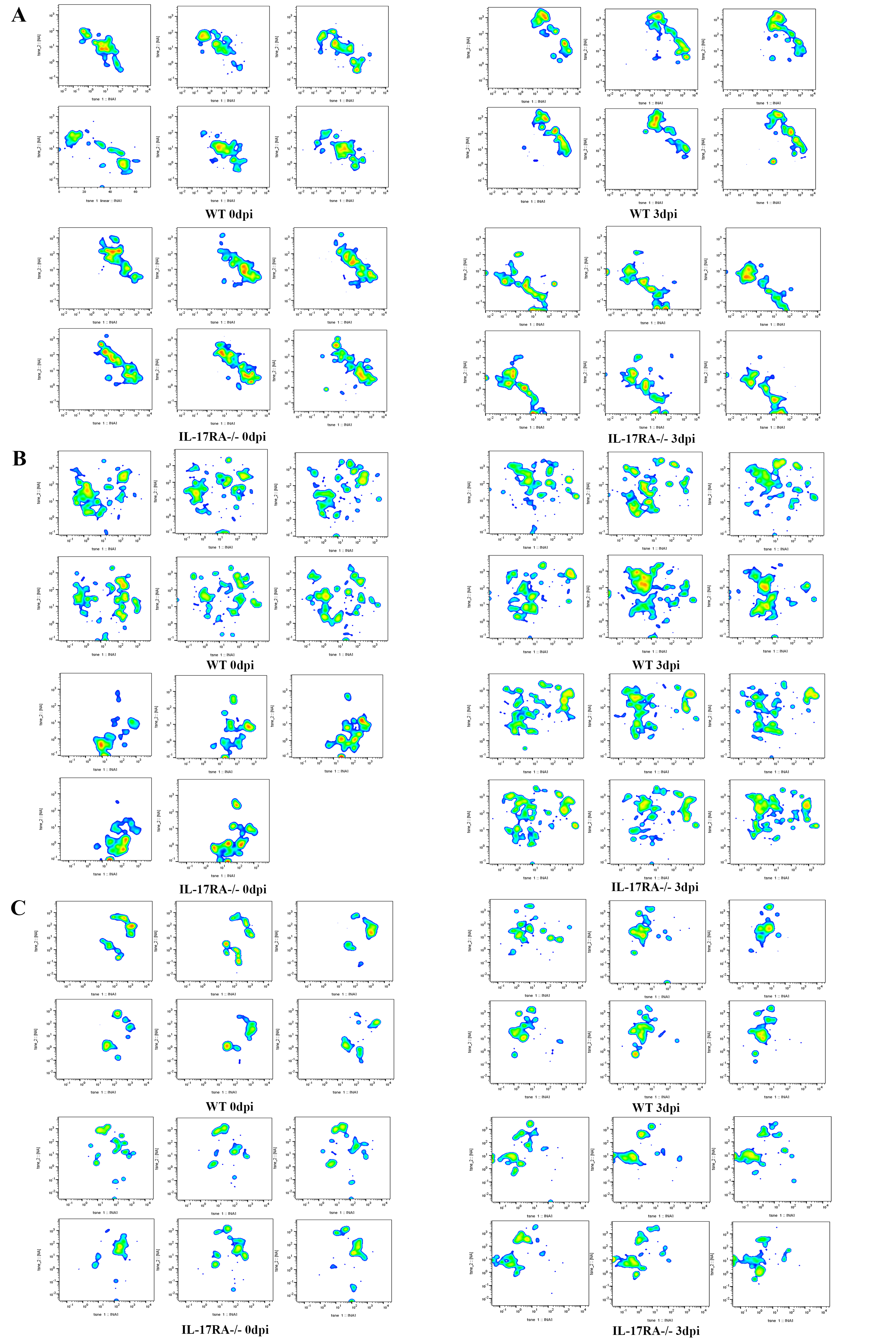

Supplement: Supplementary file 9 [file Image_6.tif]
